# Archaeological evidence that a late 14th-century tsunami devastated the coast of northern Sumatra and redirected history

**DOI:** 10.1073/pnas.1902241116

**Published:** 2019-05-28

**Authors:** Patrick Daly, Kerry Sieh, Tai Yew Seng, Edmund Edwards McKinnon, Andrew C. Parnell, Ardiansyah, R. Michael Feener, Nazli Ismail, Nizamuddin, Jedrzej Majewski

**Affiliations:** ^a^Earth Observatory of Singapore, Nanyang Technological University, Singapore 639798;; ^b^Private address, Bogor 16810, Jawa Barat, Indonesia;; ^c^Insight Centre for Data Analytics, Hamilton Institute, Maynooth University, County Kildare W23 F2K8, Ireland;; ^d^Informatics Department, Syiah Kuala University, Banda Aceh 23111, Indonesia;; ^e^Centre for Islamic Studies, University of Oxford, OX3 0EE Oxford, United Kingdom;; ^f^Department of Geophysics, Syiah Kuala University, Banda Aceh 23111, Indonesia

**Keywords:** tsunami, Sumatra, Aceh, postdisaster recovery, hazards

## Abstract

Archaeological evidence shows that a predecessor of the 2004 Indian Ocean tsunami devastated nine distinct communities along a 40-km section of the northern coast of Sumatra in about 1394 CE. Our evidence is the spatial and temporal distribution of tens of thousands of medieval ceramic sherds and over 5,000 carved gravestones, collected and recorded during a systematic landscape archaeology survey near the modern city of Banda Aceh. Only the trading settlement of Lamri, perched on a headland above the reach of the tsunami, survived into and through the subsequent 15th century. It is of historical and political interest that by the 16th century, however, Lamri was abandoned, while low-lying coastal sites destroyed by the 1394 tsunami were resettled as the population center of the new economically and politically ascendant Aceh Sultanate. Our evidence implies that the 1394 tsunami was large enough to impact severely many of the areas inundated by the 2004 tsunami and to provoke a significant reconfiguration of the region’s political and economic landscape that shaped the history of the region in subsequent centuries.

## Significance

We demonstrate that a tsunami in the late 14th century CE destroyed coastal sites along a critical part of the maritime Silk Road and set in motion profound changes in the political economy of Southeast Asia. Our results provide a precise chronology of settlement and trade along a historically strategic section of the Sumatran coast and are robust physical evidence for the rise of the Aceh Sultanate. Tragically, coastal areas impacted by the late 14th century tsunami were devastated by the 2004 Indian Ocean tsunami. This makes our findings relevant to debates about hazard mitigation and risk reduction. This example shows that archaeological, historical, and geological data are relevant in discussions about the long-term sustainability of communities exposed to geological hazards.

One reason why the tragedy of the 2004 Indian Ocean tsunami was so great is that no one saw it coming. Devastated communities had no historical precedent to alert them to the possibility of a great tsunami. Moreover, the common wisdom in the scientific community was that subduction megathrusts that lacked an historical record of producing great earthquakes were indeed not seismogenic or tsunamigenic (1).

**Fig. 1.**
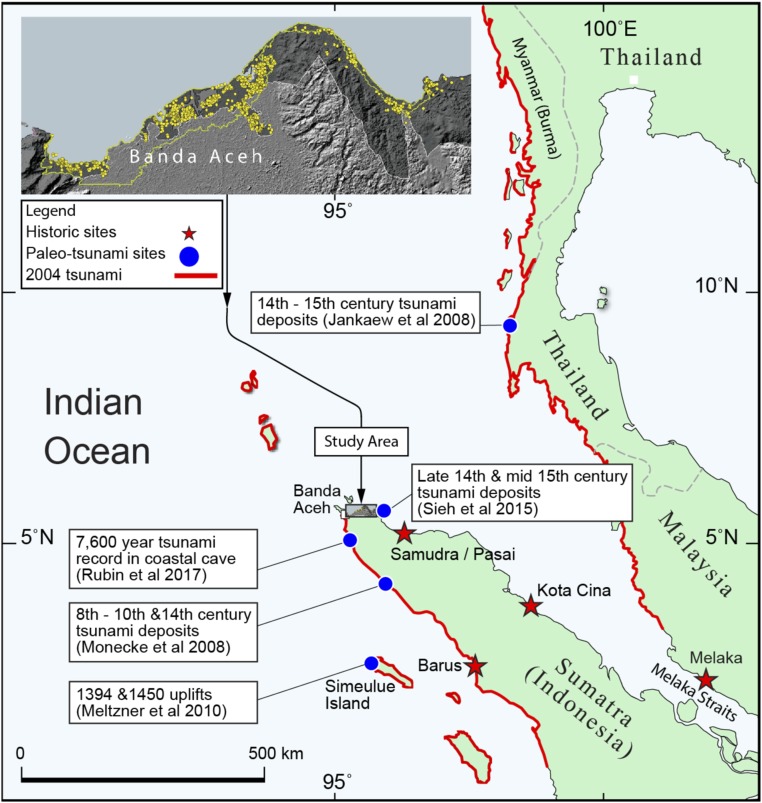
Recent paleotsunami and paleogeodetic research shows a long history of tsunami impacting Sumatra and the Malay Peninsula, with multiple sources supporting a late 14th century predecessor to the 2004 Indian Ocean tsunami (5, 8, 9, 12). The red line indicates areas inundated by the 2004 Indian Ocean tsunami (source of the coastal extent of the 2004 tsunami from the United Nations Office for the Coordination of Humanitarian Affairs). (*Inset*) Our coastal survey is between Banda Aceh and the site of Sieh et al. (14). All archaeological sites found and documented by our survey appear as yellow dots. Image courtesy of Y. Descatoire (Earth Observatory of Singapore, Singapore).

To the contrary, the great 2004 earthquake and tsunami could have been anticipated if paleotsunami and paleogeodetic research had occurred in the years before the disaster. Following the 2004 tsunami, a flurry of such research greatly changed what we know about the long-term history of tsunami in the eastern Indian Ocean (2–14). Discoveries of sand deposits in swales behind Thai and Acehnese beach ridges revealed the predecessor of the 2004 event most likely occurred between about 1300 and 1500 CE (5, 9) (Fig. 1). Paleogeodetic work, based upon coral microatolls above the southern end of the 2004 rupture, constrained precisely the dates of two plausibly seismogenic and tsunamigenic uplifts within this broad period: 1394 ± 2 CE and 1450 ± 3 CE (8). During our initial exploration for tsunami destruction a few years ago, we found compelling stratigraphic and archaeological evidence for tsunami destruction of an archaeological site soon after the 1366 ± 3 CE date of construction of a small coastal structure about 25 km east of Banda Aceh (14) (*SI Appendix*, Fig. S1).

These discoveries led us to wonder whether and to what degree historical tsunami might have affected settlement and trade along the same coasts that were devastated in 2004. Significant effects seemed plausible, as early Arabic, Chinese, and Malay documentary sources point to the importance of the coast of Aceh as a locus of inter-Asian commerce and culture since at least the ninth century CE (15–19). It is therefore likely that the coast was populated around the time of the 1394 and 1450 uplifts and affected by any tsunami caused by them. However, the scarcity of archaeological evidence has prevented robust assessment of the human impact of the events in ∼1394 or ∼1450.

In this paper, we report the results of a systematic archaeological survey over a 40-km section of the northern Sumatran coast around Banda Aceh. We located and documented surface scatters of archaeological material to determine if the 1394 and 1450 tsunami impacted coastal communities, how powerful these tsunami might have been, and how impacted communities recovered over time. Because these materials represent many centuries of occupation, this work also illuminates the development of coastal settlements through most of the past millennium along this important stretch of the medieval maritime Silk Road and represents a major archaeological landscape survey in northern Sumatra.

## Results

### Analysis of Ceramic Data.

**Fig. 2.**
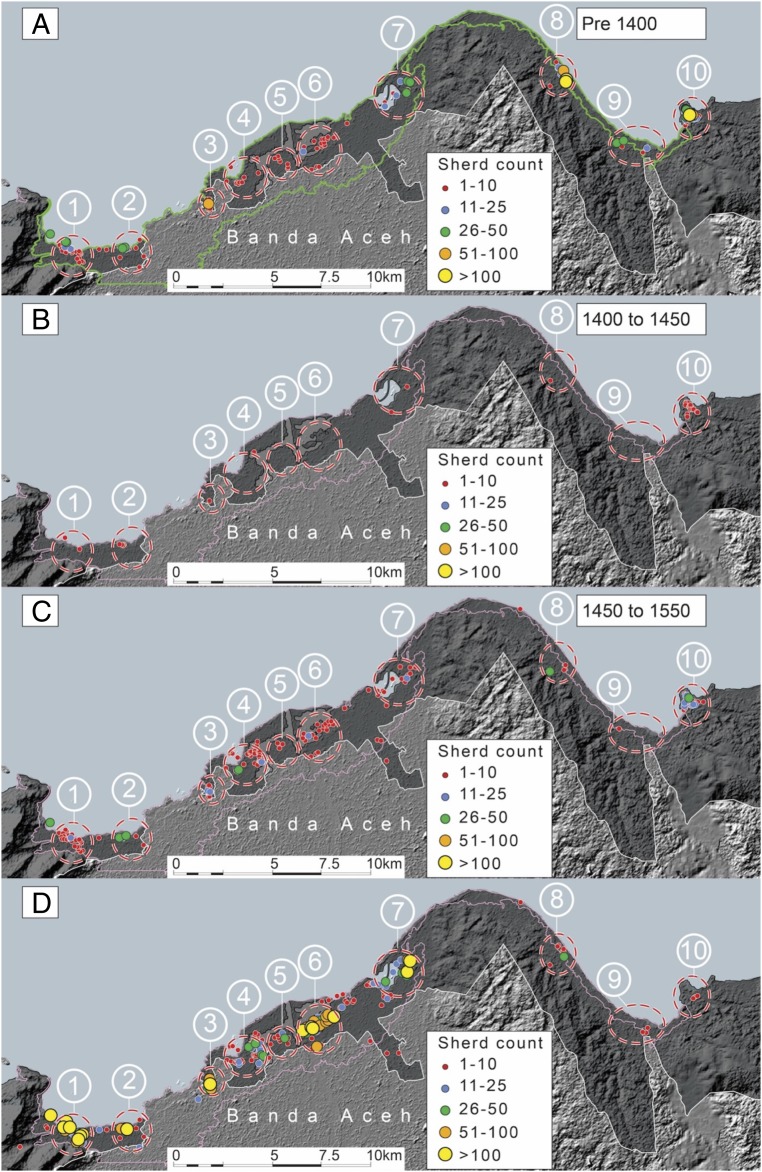
Concentrations of ceramic sherds along the Aceh coast during four intervals between the 11th and 17th centuries CE. The darker gray-shaded area indicates our survey area. (*A*) During the four centuries before 1400, ceramic use occurred in 10 clusters along the northern coast of Aceh. Abundances in clusters 8 and 10 are particularly high. Ellipses indicate rough boundaries of the clusters and appear for reference in the three subsequent figures. The green and pink lines indicate the inundation limit of the 2004 tsunami. (*B*) Dramatic reduction in sherds occurred during the period between 1400 and 1450. Moreover, Table 1 shows that 56 of the 70 sherds produced during this interval come from elevated cluster 10. (*C*) Sherd abundance began to increase between 1450 and 1550, primarily within the same clusters that existed before 1400. (*D*) Between 1550 and 1650, sherd abundance increased dramatically in the western clusters and declined nearly to zero in cluster 10. Expansion of sites beyond the limits of the 10 pre-1400 clusters began to occur in the western half of the region. *SI Appendix*, Fig. S2 shows that the distribution of settlements between 1650 and 1800 is a continuation of the pattern depicted in Fig. 2. Image courtesy of Y. Descatoire (Earth Observatory of Singapore, Singapore).

**Fig. 3.**
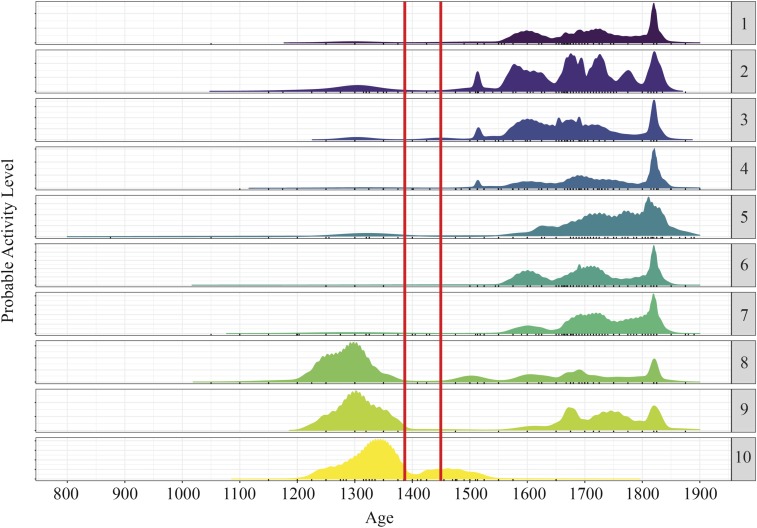
Plot of the activity levels for each cluster as a function of time, with the *x* axis showing years CE. A shift from pre-1394 tsunami dominance by clusters 8–10 to the posttsunami dominance by clusters 1–7 is clear. The peaks represent periods of activity based upon the date ranges of ceramics analyzed for each cluster. We interpret these peaks as periods of high relative activity. The patterns show that activity before the 1394 tsunami was concentrated in clusters 8–10. There is an immediate drop in activity across the survey area following the tsunami, and then a slow resumption of activity starting between the late 15th and mid-16th century. The main concentrations of posttsunami activity occur in clusters 1–7, representing a shift in settlement and trade activity. A more detailed description of the statistical method used to obtain this plot is provided in *Methods* and *SI Appendix*, *Supplementary Information Text*.

Here, we present analysis of over 30,000 pieces of trade ceramics. Based upon dated reference collections, we were able to assign all of these sherds a date range and provenance, which allows us to identify, date, and characterize areas of past human settlement. Our data reveal 10 distinct clusters of trade-ceramic sites before the 1394 tsunami (Fig. 2 and Table 1). This implies 10 geographically distinct settlements existed along this stretch of the coast. The rise of these settlements began in about 1200 CE, as evidenced by the rise of activity levels through the 13th century (Fig. 3).

**Table 1.** Quantities of confidently dated ceramic sherds for 10 geographic clusters

	Total count				
Period	Pre-1400	1400–1450	1450–1550	1550–1650	1650–1800
Cluster 1	256	2	84	1,607	2,737
Cluster 2	169	6	243	454	774
Cluster 3	76	1	107	592	727
Cluster 4	112	2	208	591	1,738
Cluster 5	125	0	5	65	635
Cluster 6	71	0	45	1,157	2,081
Cluster 7	220	2	22	587	2,426
Cluster 8	457	1	41	48	92
Cluster 9	197	0	2	2	89
Cluster 10	2,188	56	210	7	0
Total	3,871	70	967	5,110	11,299

The data show the counts of ceramic sherds at the 10 clusters that most likely date from before the 1394 tsunami. There is a notable reduction in quantity after the tsunami and, over the next two centuries, a dramatic increase in material in clusters 1–7 and a decrease in clusters 8–10.

Other first-order patterns in the data are as follows. Most ceramics before the 15th century, including fine ware that implies affluence, occur in the east, in clusters 8 and 10 (Fig. 2*A*). In contrast, most ceramics produced after the 15th century occur in the west, in clusters 1 through 7 (Fig. 2 *C* and *D*). Notably, there is a dearth of ceramics from the first half of the 15th century, between the times of the two tsunami (Fig. 2*B*). Moreover, almost all of the ceramics from that period occur at cluster 10, which is the only settlement that sits on a hill, well above the height of the 2004 tsunami.

On its face, this pattern supports the hypothesis that the 1394 tsunami destroyed all low-lying coastal settlements, but not the elevated settlement in cluster 10, and that recovery occurred slowly over several centuries, predominantly in the western settlements. Beyond this broad-brush interpretation, however, both the archaeological data and related historical documentation allow the more rigorous analysis and complex interpretations that follow.

### Settlements Before 1400.

The distribution of dated ceramics shows 10 distinct clusters of activity that began in the 13th century (Figs. 2*A* and 3). Over 73% of the pre-1400 ceramics came from clusters 8–10, along the eastern half of the surveyed coast (Table 1 and *SI Appendix*, Fig. S3). Cluster 10 alone yielded 56% of the total. The six western clusters produced the remaining 27%.

In addition to its large quantity of sherds, cluster 10 contained a more diverse range of ceramic vessel types than the other clusters (*SI Appendix*, Tables S1 and S2). Moreover, these were obtained from a wider range of production centers, including China, India, Syria, Thailand, and Vietnam (*SI Appendix*, Table S3 and Dataset S1). Cluster 10 also yielded more “high-class” fine wares and more sherds from large shipping jars than all of the other clusters combined (*SI Appendix*, Fig. S5 and Table S1). This implies that cluster 10 was the most prosperous of the clusters and well connected to regional trade networks.

In contrast, the types of the ceramics recovered from clusters 1 through 9 indicate a predominance of domestic uses: items for storage and cooking, as well as other kitchenware (Dataset S1). While we recovered some high-status fine-ware material from these settlements, quantities were far less than those found in cluster 10 (*SI Appendix*, Fig. S5 and Table S1). Hence, we conclude that these sites were smaller, less prosperous settlements.

We propose that archaeological material in cluster 10 is part of the remains of Lamri, a trading site on the medieval maritime Silk Road. [This paper is part of a wider investigation of the history of human settlement, maritime trade, and environment in the Straits of Melaka region based upon the extensive data our team collected in Aceh.] Although “Lamri” is mentioned in early Chinese-, Arabic-, and Malay-language documentary sources, its location had not previously been verified. Our data imply that Lamri was on the headland. Thus, this elevated location was the dominant center of economic and political power along this stretch of coast before the 1394 tsunami.

### Settlements Between 1400 and 1450.

Ceramic abundance and distribution for the five decades between 1400 and 1450 differ markedly from the pre-1400 pattern (Figs. 2*B* and 3 and Table 1). The most important difference is that none of the settlements yielded an abundance of sherds and some yielded none. In contrast to the over 3,800 pre-1400 sherds, this half-century yielded a mere 70. Moreover, 56 of these sherds came from cluster 10.

What is clear from this concentration is that only Lamri maintained external trade contacts through the first five decades of the 15th century. Among its sparse ceramics are Thai, Burmese, and Chinese varieties (*SI Appendix*, Fig. S4 and Table S3). Four sherds found at cluster 10 are from Imperial Chinese tribute ceramics produced during this period, most likely brought as gifts by the Chinese Admiral Zheng He to local rulers at Lamri in 1405, 1408, and/or 1416, landings that are known from Chinese archives.

### Settlements Between 1450 and 1550.

Activity started to resume in the western clusters by the late 15th to early 16th century, picking up substantially by the middle of the 16th century (compare Figs. 2 *B* and *C* and 3 and Table 1).

Significant differences between the pre-1400 and 1450–1550 settlements exist in both the quantity and types of ceramics used. This implies significant economic and societal changes along this stretch of the Sumatran coast between these periods. The eastern settlements (clusters 7–10) saw their share of ceramics drop from a high of 76% of the pre-1400 material to only 26% of the 1450–1550 material (Table 1). This is especially pronounced for Chinese ceramics, as we recovered only 13 sherds of Chinese ceramics from clusters 8–10 produced during this period. This is a remarkable difference from the pre-1400 distribution, during which clusters 8 and 10 contained the largest amounts of Chinese material.

Conversely, clusters 1–4 account for over 70% of the ceramics from this period, including a significant amount of Chinese trade ceramics (Table 1). Importantly, these include highly distinctive “blue and white” fine-ware porcelain that was exported in large quantities from kilns in Jingdezhen, China between 1506 and 1521. This material was popular throughout Southeast Asia and is likely to be present at any site engaged in trade with China during this period. We recovered 289 pieces of Jingdezhen material from clusters 1–7, but only 12 pieces from clusters 8–10 (*SI Appendix*, Fig. S6 and Table S2). The changing distribution of ceramics strongly implies that by the first decade of the 16th century, an emerging economic power in the western clusters 1–4 had replaced the pre-tsunami concentration of trade and power in clusters 8–10, including the erstwhile settlement of Lamri on the headland.

### Settlements Between 1550 and 1650.

In western clusters 1–7, ceramic sherds produced from 1550 to 1650 are dramatically more abundant than sherds from the previous century (compare Fig. 2 *C* and *D*, *SI Appendix*, Fig. S3, and Table 1). In contrast, concentrations at clusters 8 and 9 remain low, and at cluster 10, concentrations decline to near zero.

The western clusters also show a greater diversity of vessel types from a wider range of production centers. Most of the high-class fine wares during this period are from clusters 1–7 (*SI Appendix*, Fig. S5 and Table S1). Starting around 1550, regional trade networks were flooded by Chinese ceramics from production centers in Zhangzhou, making this material an important indicator of engagement with interregional trade. We recovered 3,653 sherds of this material, with 3,600 of the sherds coming from clusters 1–7, and only one sherd recovered from cluster 10 (*SI Appendix*, Fig. S6 and Table S2). Thus, it is clear that these western settlements continued to grow in affluence over the late 16th and early 17th centuries, cementing their status as the new locus of trade on this coast. We were not able to conduct our survey in the modern dense urban areas of Banda Aceh, which overlie the historical core of the Aceh Sultanate (as illustrated by the gap between clusters 2 and 3 in the survey zone in Fig. 2). Therefore, we take our evidence for settlement in the western clusters to be a minimum estimate for human activity, as it may well be that this gap was also becoming densely settled.

Material recovered from clusters 8 and 9 show small-scale resumption of settlement, with their respective assemblages dominated by coarse wares, largely of a domestic nature (Fig. 2*D*, Table 1, and *SI Appendix*, Table S3). In contrast to the continued growth of the western settlements, the very low sherd numbers at clusters 8 and 9 indicate that these settlements had been all but abandoned. Moreover, ceramic sherds from cluster 10 are also sparse (Table 1). Notably, the absence of Zhangzhou ceramics establishes that cluster 10 had been abandoned as a habitation and trade site by 1550 (*SI Appendix*, Fig. S6 and Table S2).

Defensive earthen and stone fortifications built on the headland in cluster 10 between 1490 and 1650 CE suggest that the new polity emerging in the western seven clusters established defenses on the ruins of the headland settlement during this period to ward off European colonial antagonists and/or competitors during this period, to secure access to fresh water, and/or to lock in the growing port’s trade monopoly (14). This is consistent with the disappearance of mentions of Lamri in written sources after about 1550, by which time there is tangible evidence of the establishment of the Aceh Sultanate as the new center of economic prosperity and military power in the region (15).

### Gravestones.

Here, we present the spatial distribution of over 5,000 carved stone grave markers that we were able to date based upon style and inscriptions. Constraints on the dates of the thousands of gravestones are not as tight as those on the ceramics. Based upon comparison with dated gravestones from the region, we are able to divide the gravestones into two crude periods relevant for this analysis: those set within the 15th century and those set from the mid-16th through early 19th centuries (*SI Appendix*, Table S4).

**Fig. 4.**
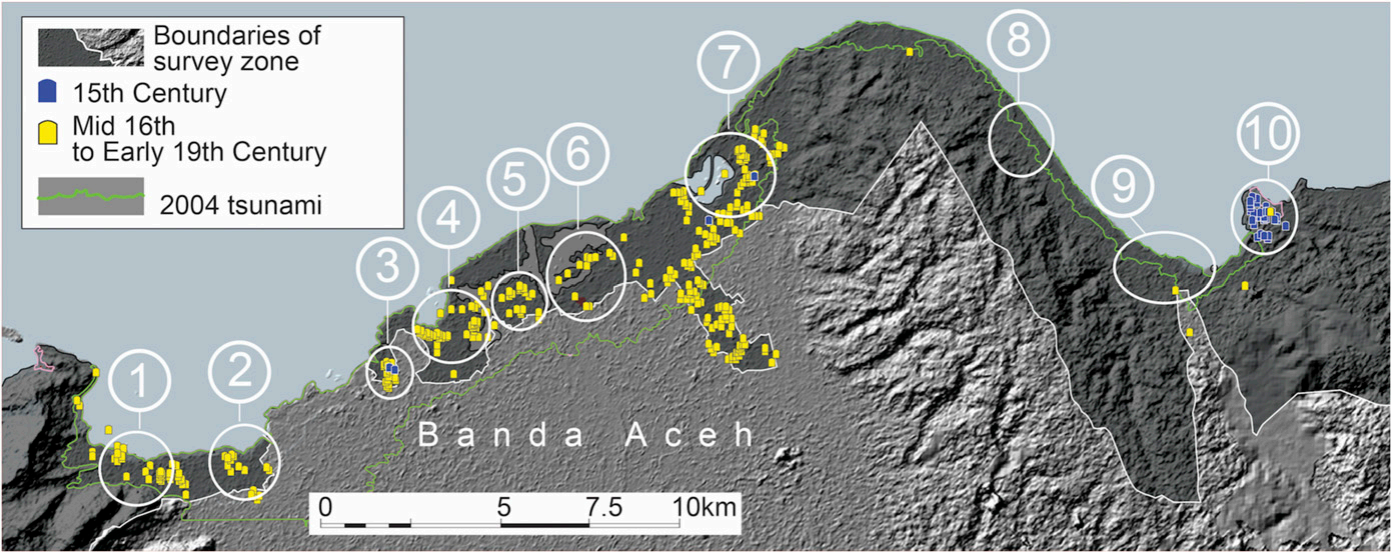
Distribution and types of gravestones show that styles of Muslim funerary markers changed significantly over the 15th and 16th centuries. Almost all graves that we believe date to the 15th century (in blue) are located on the elevated headland in cluster 10. The yellow markers are all types that are associated with the Aceh Sultanate and date from the mid-16th century onward. Image courtesy of Y. Descatoire (Earth Observatory of Singapore, Singapore).

The oldest datable gravestones within our surveyed section of the Aceh coast date to the first half of the 15th century, on the basis of both inscriptions and stylistic comparisons with published examples of similar forms. Almost all gravestones that we can date to the 15th century are in cluster 10 (Fig. 4). This includes 116 ornately carved gravestones locally known as *plang pleng* (*SI Appendix*, Fig. S7 and Table S5). These are part of a highly distinctive early Muslim funerary tradition found only on the Aceh coast. Published dated examples of *plang pleng* gravestones, including several from our survey zone, place this style between the years 1419 and 1490 CE (20).

We also found a number of gravestones in cluster 10 that have clear stylistic similarities to known examples from Pasai that date to between 1416 and 1483 CE (*SI Appendix*, Figs. S8 and S9 and Table S5). Pasai was a major Muslim polity of the late 13th to early 16th centuries on the Sumatran side of the Straits of Melaka near the present-day coastal town of Lhokseumawe, 210 km east-southeast of our survey area (Fig. 1). The presence of the 15th century graves almost exclusively in cluster 10 reinforces its continued use in the century following the 1394 tsunami.

In contrast, almost all gravestones dating from the mid-16th century onward are stylistically distinct from those set mostly at Lamri in the 15th century and are in clusters 1 through 7 (Fig. 4). These comprise highly distinctive types of gravestones associated with the Aceh Sultanate that started to appear in the mid-16th century. These *batu Aceh* gravestone types exist across Muslim Southeast Asia, with notable concentrations in Sumatra, peninsular Malaysia, and southern Thailand (21, 22). Our record of 1,598 *batu Aceh* stones that most likely date between 1550 and 1800 within the western clusters (clusters 1–7) affirms historical records that indicate this stretch of the coast was the historical center of the Aceh Sultanate (Fig. 4 and *SI Appendix*, Figs. S12–S15 and Tables S4 and S5). The scarcity of these *batu Aceh* types in clusters 8, 9, and 10 implies that the entire eastern side of our survey zone, once the dominant maritime port polity along this stretch of coast, had become a neglected hinterland during the ascendancy of the Aceh Sultanate.

## Discussion

### Summary of Findings.

The principal findings from our systematic landscape archeological survey of this 40-km-long section of the northern coast of Aceh are these:

• Ceramics produced from the 11th through 14th centuries CE reveal 10 distinct settlement clusters. The easternmost of these, cluster 10, situated on an elevated headland, was the most prosperous and had extensive international trade connections.
• Between 1400 and 1450, sherd numbers plummeted at all 10 clusters, in half of the clusters to less than 10 fragments. Most of the sherds dating to this period come from cluster 10, the only settlement that rises more than a few meters above sea level. While activity measured by number of sherds dropped considerably, cluster 10 clearly retained connections to its international trading partners over this period. Moreover, the presence there of early 15th-century Chinese Imperial ware strongly implies that we can identify the headland settlement as the major center of Lamri, a toponym mentioned in Chinese and Arabic accounts of medieval maritime trade. Continued occupation of cluster 10 throughout the 15th century is further supported by gravestones dated to this period within the cluster.
• The absence of the highly distinctive Jingdezhen ceramics produced between 1506 and 1526 at cluster 10 indicates that the headland settlement was no longer trading with China by the beginning of the 16th century. This is consistent with its disappearance from historical records from that time. In marked contrast to earlier imports, the presence of these distinctive ceramics in the western clusters demonstrates that the nature of trade with China had altered. We propose that this shift was a significant part of the formation of the Aceh Sultanate in the early 16th century.
• From ∼1450 through ∼1650, sherd numbers at sites along the western section of the coast, within and in the vicinity of clusters 1 through 7, increased, with a dramatic jump in the quantity of material recovered dating between 1550 and 1650. Almost all gravestones that postdate the 15th century are located on the western side of the study zone as well, with very few found in clusters 8–10. The nature of the sherds and gravestones is consistent with historical sources, which mark this as the peak period of economic and military power of the Aceh Sultanate.

### Interpretation of Findings.

It is clear from our data that something provoked a major depopulation of the northern coast of Aceh around 1400. The data also make clear that this was followed by a slow repopulation and significant changes in the political economy of the region between 1450 and 1550, which involved abandonment of the previous principal trading port on the headland in favor of what became the Aceh Sultanate, farther west along the coast.

One might propose a cause not related to a devastating tsunami, such as an externally driven change in trade patterns or a local political conflict. Among these alternative hypotheses, the most credible is the disruption of ceramic trade by a series of Chinese Imperial production and export bans between 1371 and 1529 known as the Ming Gap. This is known to have caused a reduction in the quantity of Chinese ceramics imported during this long period.

We know, however, that despite the ban, distinctive Chinese Imperial ceramics continued to be gifted to rulers within the Chinese sphere of influence throughout the 15th century. Furthermore, data from shipwrecks in Southeast Asia show that Thai, Burmese, and Vietnamese ceramic production centers increased exports by about 1400 to fill the vacuum left by the Chinese withdrawal from Southeast Asian markets (23).

Our data allow a test of the hypothesis that the Ming export bans are the primary cause of the dramatic changes in the geography of settlements that occurred along the Aceh coast between 1400 and 1650:

• Of the 10 clusters, only cluster 10 is positioned above the run-up elevation and outside of the inundation zone of the 2004 tsunami. This headland settlement contains most of the imported ceramics dating to the period 1400–1450, including special Chinese tribute wares and material from Thai, Burmese, and Vietnamese production centers that filled the vacuum created by the Ming export ban. Additionally, nearly all of the gravestones that we can date to the 15th century are located on the headland in cluster 10. This is an indication of continued settlement and significant economic prosperity throughout the 15th century, a factor quite independent of fluctuations in trade of Chinese ceramics.
• In the low-lying clusters 1 through 9, it is not just Chinese ceramics that are absent between 1400 and 1450, but also ceramics from non-Chinese kilns that filled the market need elsewhere in the region beginning about 1400. Of the 37 Southeast Asian sherds that we can date with confidence between 1400 and 1450, 35 were recovered from cluster 10. If the other settlements were active during the first half of the 15th century, we would expect to recover higher quantities of the Thai, Burmese, and Vietnamese ceramics that substituted for Chinese material.
• The Ming ban started in 1371, but our combination of gravestones, Chinese Imperial tribute ceramics, and imported ceramics from Thailand and Burma shows that cluster 10 continued to be occupied throughout the entire 15th century in what is likely to have been an elite merchant community. If the Ming ban was responsible for the changes presented in this paper, the changes would have started in the late 14th to early 15th century. The endurance of the headland settlement as a prominent site for over a century after the ban started suggests the transformation of power dynamics along the coast had other causes.
• There is no clear reason why the Ming export ban would lead to a relocation of the center of economic power from Lamri to the western clusters in the early 16th century.

These observations favor the interpretation of the archaeological data as reflecting the occurrence of a large tsunami, consistent with the geological evidence of tsunami inundation and destruction at two sites near sea level on the Lamreh headlands soon after 1366 ± 3 CE (14), tsunami inundation in western Aceh and of coastal sites in Thailand and western Aceh between about 1300 and 1500 CE (5, 9), and evidence of sudden uplift on Simeulue island at the southern end of the 2004 rupture in both 1394 ± 2 CE and 1450 ± 3 CE (8) (Fig. 1).

The archeological data show that the 1394 event, like the 2004 tsunami, destroyed all of the low-lying coastal settlements along this section of the coast. The only surviving settlement was on the Lamreh headland, mostly well above the run-up height of even a tsunami as great as that of 2004. We speculate from the material culture recovered that while reduced in stature, Lamri must have maintained sufficient prominence to maintain contact with regional traders and serve as a landing point for Zheng He’s fleet in the early 15th century. The destruction of the other nine settlements strengthens the argument that the 1394 event was large and possibly on par with the 2004 tsunami. Previous indications of its size and impact are less compelling: inundation of two sites on the Lamreh headland that are within 3 m of sea level and uplift of coral microatolls at just one point above the southern end of the 2004 megathrust rupture. Uplift there was a third as high in 1394 as it was in 2004 (1.5 m versus 0.5 m).

Uplift at that locality in ∼1450 was about 3.0 m, about twice that of 2004. As yet, however, we have no compelling evidence that a tsunami had a major impact on historical settlements along this reach of the Aceh coast in ∼1450. Our data do not argue, however, against its occurrence. The best direct evidence for the 1450 tsunami remains the younger of the two coral-rubble beds at the Lubhok Bay site on the Lamreh headland (14). In terms of impact on coastal settlements, it is possible either that the mechanics of the 1450 rupture did not generate a tsunami as powerful as the 1394 and 2004 events or that there was a tsunami but that recovery from the 1394 event had not yet begun in earnest by 1450, so there would have been little human infrastructure to destroy in the affected areas. Further research is needed to clarify the nature of the 1450 event.

What is clear from our archeological survey is that the 1394 tsunami helped set in motion a major change in trade and power dynamics along this stretch of coast. Somewhat ironically, the elevated settlement of Lamri, which survived the tsunami, was largely abandoned starting no later than the early 16th century, just as a new power center emerged in a collection of sites that had been destroyed only decades earlier.

Let us speculate as to why this happened. The early 16th century appearance of several types of gravestones in cluster 4 that share characteristics with those in Pasai suggests that recovery was not led by tsunami survivors and neighbors living immediately outside of the inundation zone, who might have slowly moved back toward the coast to rebuild (*SI Appendix*, Fig. S10). Rather, we believe the destruction caused by the tsunami provided an opportunity for people from further afield, including Pasai, to move into the area and set up a trading port in prime locations on vacated land. One reason this scenario is appealing is that it could draw on the desire of Muslim traders to move away from locations closer to Melaka, which the hostile Portuguese had conquered in 1511.

Converging around what is now Banda Aceh would have shifted the interests of Muslim merchants who had previously traded through Pasai to a new site farther from this troublesome new center of European competition and power in the region. Fellow traders in Pasai might also have been motivated to join the Acehnese by environmental factors, including the silting up of their harbor that was occurring throughout the latter half of the 15th century (24). In light of these significant historical shifts along the Straits of Melaka, the stretch of Sumatran coast depopulated by the 1394 tsunami appears to have been increasingly attractive to Muslim traders in the region. The new port polity that was established there in the early 16th century most likely was the start of what became the Aceh Sultanate, which eclipsed Lamri, the earlier established settlement on the headland, to become the dominant economic and military power in the region for the next two centuries.

## Conclusion

We now have knowledge of two occasions when settlements along the northern Sumatran coast were devastated by great tsunami: one in ∼1394 and another in 2004. Following the 1394 event, resettling and reestablishment of long-distance trading ports in the inundation zone took almost a century and involved newcomers from areas that were not impacted by the tsunami. After the 2004 tsunami, a major international reconstruction effort mobilized resources to rebuild and repopulate destroyed coastal areas in less than a decade (25–28). In both cases, the end result was the same: large numbers of people living in an area exposed to significant tsunami hazard and risk.

From a scientific perspective, continuing to reside in areas exposed to hazards is puzzling and often frustrating, especially when the scale of human loss is still fresh in mind. When viewed from a longer historical perspective, however, it is our default setting. The historical and archaeological records are filled with examples of cities destroyed and rebuilt, only be destroyed again (e.g., refs. 1, 25, 29, 30). In the wake of the 2004 tsunami, some proposed moving coastal communities inland and creating a buffer zone to ensure the safety of future residents. However, a combination of political and economic realities, and pressure from inhabitants and members of the international humanitarian sector to rebuild quickly, resulted in rebuilding in situ. Unfortunately, it is geologically certain that these new settlements along this same stretch of coast will suffer destruction again. The only question is whether it will be sooner or later.

This less-than-optimal situation is the result of two realities. First, most communities are partial to the geography they and their forebears have known for centuries. It is important to acknowledge that the location of settlements and cities is influenced by a complex mix of reasons, from access to resources and livelihoods and proximity to transportation routes, to cultural and religious attachments to place, to the availability of new and safer land. Exceptions do exist. The self-imposed relocation to higher, inland locations of many villages along the west coast of the Mentawai Islands following the deadly Sumatran tsunami of 2010 is one. Second, the hazard that communities face unleashes itself only very, very infrequently, generally never more than once every several generations. Households and societies are much more responsive to immediate and short-term hazards, and the associated risks, than they are to longer term and less likely hazards. It is difficult to imagine most stakeholders agreeing to a mass relocation based upon what might happen at an unspecified time in the very distant future. Nonetheless, the history of devastation of the Aceh coast is a powerful reminder that actions in the past matter to future generations and that the distant future does eventually arrive.

## Methods

Our work encompassed 43 villages that comprise the highlighted regions in Fig. 1, *Inset*. These regions include all villages along a 40-km section of coast, with the exception of the modern dense urban areas of Banda Aceh, and a transect of village running inland perpendicular to the coast to provide data on upland areas that might have extended beyond the reach of the 1394 tsunami. We discovered 1,029 sites through consulting local inhabitants and systematic field exploration. By “sites,” we mean discrete concentrations of cultural material visible on the ground surface. This material includes carved stone grave markers, structural remains, and surface scatters of ceramics. For each site, we recorded global positioning system positions, wrote site descriptions, took extensive photographs, and collected all potentially diagnostic ceramic material on the surface. We conducted no subsurface excavations, although some of the collected ceramics were brought to the surface by dredging for fish ponds before and after the 2004 tsunami.

We documented over 5,800 individual Islamic gravestones and collected nearly 52,000 ceramic sherds. Aside from providing an archaeological test of the occurrence of a tsunami, this dataset is robust evidence for the inhabitation of the Aceh coast through the past millennium.

We categorized the Muslim gravestones by morphology, ornamentation, and textual inscriptions and compared them with gravestones from elsewhere in the region that have been dated by their inscriptions (20–22, 31–36). Many of them have Arabic inscriptions, most often of Qur’anic verses and other religious formulae, and relatively few bear personal names and/or death dates. We were able to place ∼30% of the grave markers within broadly demarcated periods: one concentration within the 15th century and another within the mid-16th to early 19th centuries (*SI Appendix*, *Supplementary Information Text*).

Our team washed, labeled, and inspected every ceramic sherd. We were able to obtain information on chronology and provenance for 61% of them and used that information in this analysis. Of the ∼52,000 sherds collected, we believe just over 8,000 sherds are locally produced earthenware ceramics. As this earthenware material cannot be dated precisely using datable reference collections, we do not consider that local material here. The ceramic analysis in this paper is based solely upon imported trade ceramics.

We assigned each datable sherd a minimum and maximum date of production based upon published ceramic reference collections (e.g., refs. 23, 37–40) and the decades of experience of two of the authors (E.E.M. and T.Y.S.) in excavating and dating regional trade ceramics. Given the known rapid evolution of types and styles of trade ceramics in the region, it is reasonable to assume that use usually occurred soon after production, but it is possible, and perhaps even likely, that some small number of the sherds were broken and discarded decades after production. Date ranges vary considerably by the type of ceramic vessel; some types were produced for a very short period of time, while other types were produced for centuries.

To constrain better the likely periods of activity using the ∼30,000 date ranges provided by the ceramics analyzed in this paper, we applied a statistical model to the ceramic dataset to calculate probable activity levels for each year. The model produces estimated probability densities over time, which we treat as relative “activity levels” for each site. We produced the model using the BchronDensityFast function in Bchron in the R package (41, 42), which relies on the Mclust density estimation procedure (43, 44) to fit a mixture of univariate normal distributions to the samples. The details of our utilization of these methods are provided in *SI Appendix*, *Supplementary Information Text*.

To understand changes in the spatial distribution of the sherds over time, we grouped the ceramics into five periods: pre-1400, 1400–1450, 1450–1550, 1550–1650, and 1650–1800. These divisions allow assessment of whether either the ∼1394 or ∼1450 tsunami had any significant impact on coastal settlements. They also fit with known breaks in the production of regionally common ceramic styles.

## Supplementary Material

Supplementary File

Supplementary File
