# Supplementary material for: Archaeological evidence that a late 14th-century tsunami devastated the coast of northern Sumatra and redirected history
Source: Proc Natl Acad Sci U S A. 2019 May 28;116(24):11679–86. doi: 10.1073/pnas.1902241116 (PMC6587673; doi:10.1073/pnas.1902241116)
Supplement: Supplementary File [file pnas.1902241116.sapp.pdf]

www.pnas.org/cgi/doi/10.1073/pnas.1902241116

PNAS
www.pnas.org

Supplementary Information for:

Archaeological evidence that a late 14th-century tsunami devastated the coast of northern Sumatra and redirected history

Patrick Daly, Kerry Sieh, Tai Yew Seng, Edmund Edwards McKinnon, Andrew C. Parnell, Ardiansyah, R. Michael Feener, Nazli Ismail, Nizamuddin, Jedrzej Majewski

Corresponding author: Kerry Sieh
Email: Kerrysieh@ntu.edu.sg

This PDF file includes:

Supplementary text
Figs. S1 to S15
Tables S1 to S5
References for SI reference citations

Other supplementary materials for this manuscript include the following:

Dataset S1

Supplementary Information Text

Gravestone Chronology

We located and documented 5,863 gravestones during the survey. For this paper, we are able to fit 1,866 gravestones within a crude chronology based upon comparative analysis with dated, published gravestones from the region and in a few cases, graves from our survey area that have been dated based upon inscription (1 - 5). On this basis, we have identified 152 gravestones that most likely date to the 15th century, another 116 from the late 15th century through the early 16th century, and 1,598 from the mid-16th through the 18th century (Table S4). Many of the undated gravestones are simple river-rounded stone markers and non-descript standing slabs that are impossible to directly date. A large percentage of the rest of the currently undated stones most likely belong to the Aceh sultanate period (dating from the early-16th century), but more work is needed to refine the gravestone typology and chronology. The categories we use in this paper are sufficient for testing our hypothesis about possible changes to settlement caused by the 1394 and 1450 tsunamis.

15th Century Gravestones

We found three types of gravestones within the survey area that we believe were produced during the 15th century. There is a vernacular style of Muslim gravestone, locally known as *plang pleng*, that is seemingly unique to this section of the Aceh coast. *Plang plengs* are standing squared pillars that taper into a pyramidal apex, usually less than 1 meter in height. All known examples have ornamentation, which can include elaborate floral and/or geometric patterns, and Arabic inscriptions, generally contained within panels near the base of the gravestones (Figure S7). Several clusters of *plang plengs* were previously analyzed by scholars, with 11 containing the date of burial within inscriptions (Table S5) (1). Therefore, we propose

this style of gravestone dates from the early 15th through the late 15th century. We don't know of any *plang plengs* that have been dated later than the 15th century.

We recorded a number of standing squared pillar gravestones that that share some characteristics, but are distinct from the *plang pleng* tradition in terms of form and style. While we have not yet obtained any direct dating from the inscriptions on them, they share some characteristics with dated gravestones from Pasai (Figure S8) (1). The examples from Pasai date to 1415 and 1483-4. Based upon stylistic comparison, we feel it is likely that the examples we recorded in our survey zone also date to the 15th century, and are most likely indicative of some form of cultural connection between areas in our survey zone and Pasai during the 15th century.

We recorded a number of standing slab graves with inward curving sides. While most of these stones are blank, we recorded several that have elaborate ornamentation. The carved gravestones have very clear stylistic similarities with several dated sets of gravestones from Pasai that date between 1430 and 1440 (Figure S9) (1, 5). We therefore feel it is likely that the examples we recorded in our survey zone date to the 15th century and are further evidence for cultural connection between residents in our survey zone and Pasai.

Late 15th – Early 16th Century Gravestones

We recorded two types of gravestones that we suspect date between the late 15th through early 16th century. Because some of the dated comparative examples from Pasai span the two centuries, we have put them in a distinct chronological category that overlaps with the 15th century group. However, we have yet to find any examples of these gravestones that we can date conclusively before the late 15th century.

We recorded four thick standing slab gravestones with ornamentation and inscriptions that are virtually identical in style to gravestones from royal tombs in Pasai (Figure S10) (1). Dates from inscriptions in Pasai from stylistically similar gravestones range from 1510 to 1519. We therefore feel it is likely that the examples we recorded in our survey zone date to the early

16th century and are further evidence for cultural connection between residents in our survey zone and Pasai.

We recorded gravestones that we initially categorized as *plang pleng* grave markers based upon stylistic similarities. More detailed analysis has led us to suspect that there is a later type of *plang pleng* that dates from the late 15th through early 16th century. This is based upon several observations: 1) some *plang plengs* are much more homogenous in terms of physical characteristics than the types of *plang plengs* that have dated inscriptions; 2) the 'later' *plang plengs* are not found in association with any gravestones that are dated to the 15th century, but are found in association with graves dated to the late 15th – early 16th century (and later); and 3) we have found several unique examples of graves which seem to share characteristics of the 'later' *plang pleng* style and some of the early *batu Aceh* gravestones (Figure S11). This leads us to speculate that some types of *plang plengs* are younger than the dated *plang plengs* and fall into the period immediately before the Aceh sultanate developed. We did not include these grave sites on the map in the main text because our dating is highly circumstantial and not yet robust enough to go beyond speculation.

Mid 16th – Late 18th Century Gravestones

There are well-documented traditions of gravestones associated with the Aceh Sultanate called *batu Aceh*. These vary considerably in terms of size, shape and ornamentation and are found throughout areas that fell under the Aceh sultanate, including northern Sumatra, and the Malay/Thai peninsula (6 - 7). Several of the main styles of *batu Aceh* have been dated on the basis of inscriptions, with the main period of their production spanning the mid 16th century through the 18th century.

One of the most distinctive styles of *batu Aceh* are 'winged' slab stones, often with extensive ornamentation and inscriptions. Within this broad category we found an extensive variety of stones (Figure S12). Based upon comparison with dated published gravestones, we

consider these to date from the mid 16th century through the 18th century. Published accounts demonstrate that there are early examples of ‘winged’ slabs that date to the 15th century from Pasai (5 - 7). However, we did not identify similar stones in our survey zone, leading us to conclude that only later versions of this style of gravestone are present within our survey zone.

We recorded squared pillar stones, with elaborate bases, extensive ornamentation and multi-tier finials that are associated with the Aceh sultanate (Figure S13). Published dated examples suggest that this style dates from the late 16th century, possibly running through the 17th century (2 - 4).

We recorded vertical slab gravestones, with curved shoulders, multi-tier finials, and often elaborate ornamentation that are associated with the Aceh sultanate (Figure S14). Published dated examples suggest that this style dates from the mid to late 16th century (4).

We recorded gravestones that consist of vertical columns with six or eight edged faces, some with ornamentation. Several similar gravestones have been are associated with the Aceh sultanate (Figure S15). Published dated examples suggest that gravestones in our survey area that are of this style date from the late 16th century (3 – 4).

Ceramics Chronology

We dated the trade ceramics recovered during our survey based upon dated reference collections obtained from shipwrecks, archaeological investigations, and museum collections. We were able to assign a geographic source and date range of production to 61% of the ceramic sherds found in our survey area. Working with the ceramic data is complicated by the wide variety of date ranges for different types of ceramics. We recovered several sherds that we could date precisely to a specific year (based upon a stamped date). However, most of the sherds had date ranges that spanned decades, and in some cases, centuries.

We applied a statistical model to the ceramic dataset to calculate probable activity levels for

each year, using the BchronDensityFast function in the Bchron software package (8 – 9). The BchronDensityFast function estimates activity levels from large sets of date ranges by first sub-sampling the dates, and then using the Mclust density estimation procedure (10 – 11) to fit the dates within a normal distribution. The output of the BchronDensityFast model is an estimate, for each age period of the activity level measured as a probability density and so summing to 1. This can subsequently be interpreted as showing periods of high and low relative activity, especially when compared across different groups.

By default Bchron only accepts either radiocarbon or normally distributed dates, whereas the present data set contained dates only defined on a uniform interval. For that reason all dates were converted so that the uniform range was re-expressed as a 99% confidence interval for a normally distributed date. In general, the BchronDensityFast function can be seen as a more sophisticated density estimation method to that of the SUM functions currently implemented in OxCal (12).

Supplementary Figures

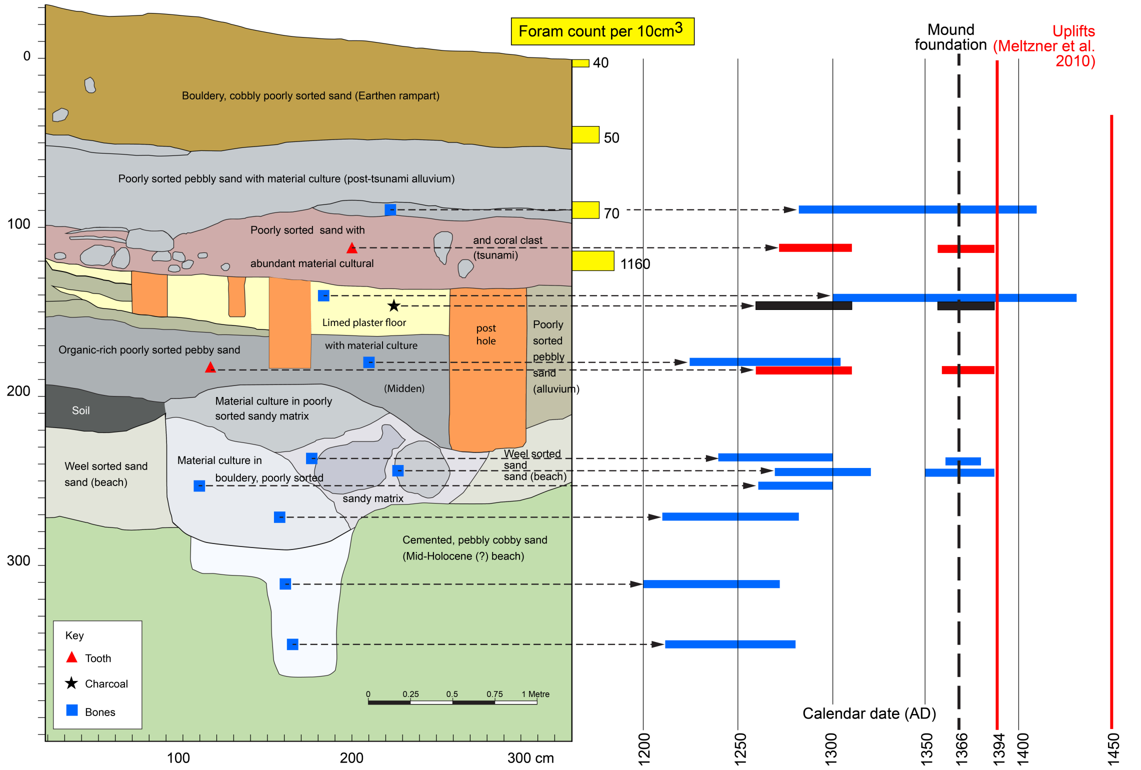

Fig. S1. Exposure of cultural layers and natural deposits in a beach cliff below the elevated headland in Cluster 10 (Lamri) revealed two centuries of occupation before destruction of a small structure by a tsunami soon after 1366±3 C.E. A 14th-century lime plaster floor (yellow) covers a 13th- to 14th-century rubbish pit (gray). A layer rich in foraminifera tests (a form of marine microfossil) and coral (pink) overlies the floor, providing evidence of marine overwash consistent with a tsunami. The uppermost layer is a man-made earthen ridge built parallel to the cliff. Blue squares, red triangles, and the black star correspond to samples with radiocarbon ages (black horizontal lines) at corresponding dates on right. The red lines indicate the two uplift events documented by Meltzner et al 2010 (13). From Sieh et al 2015 (14).

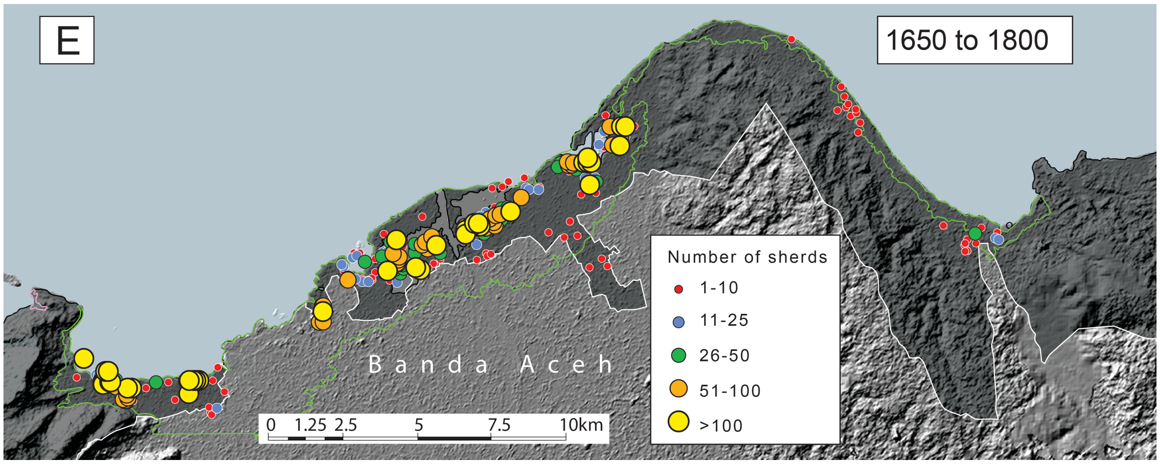

Fig. S2. Distribution and abundance of ceramics for the period 1650-1800. The data show a continuation of the trend in Figure 2 toward increasing density of settlement along the western side of the survey zone. The apex of the Aceh sultanate occurred during this time period.

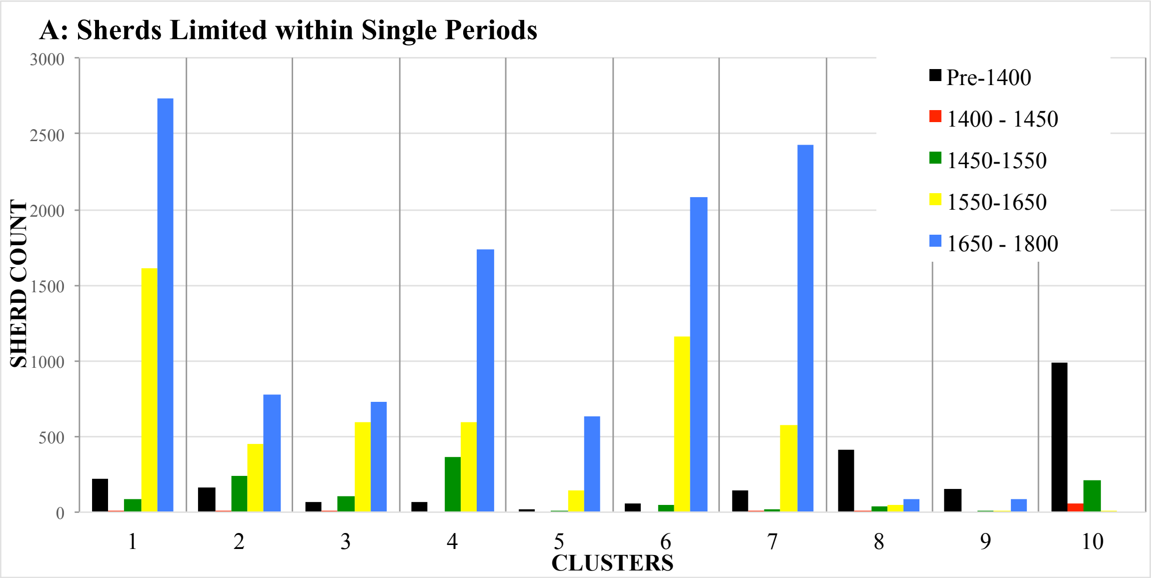

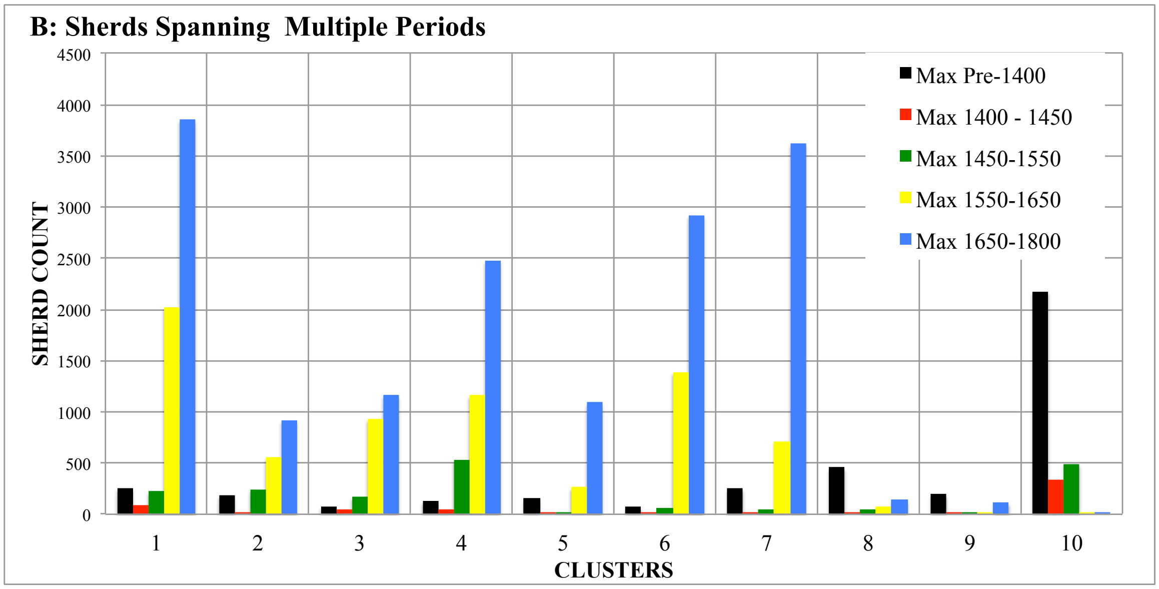

Fig. S3. Sherd count by period and cluster. *A*. Counts only of material that was definitely produced within a single time period. *B*. Counts of all material, including sherds for which the production date can range across more than one of the time periods. These materials are counted in each of the periods during which they might have been produced. Figure S3B thus gives a maximum limit for sherd counts for each period.

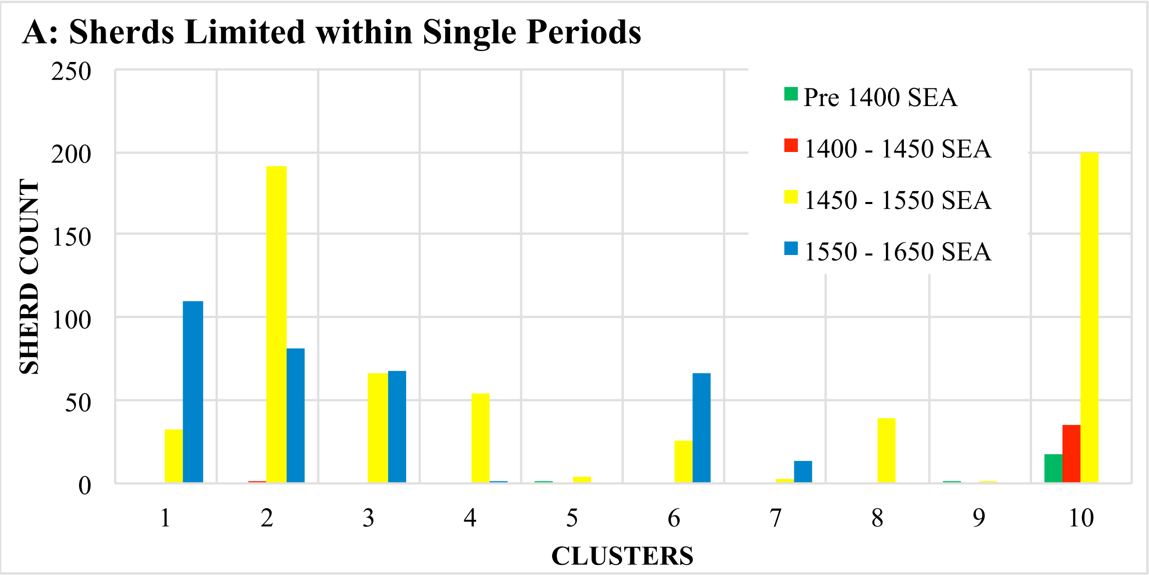

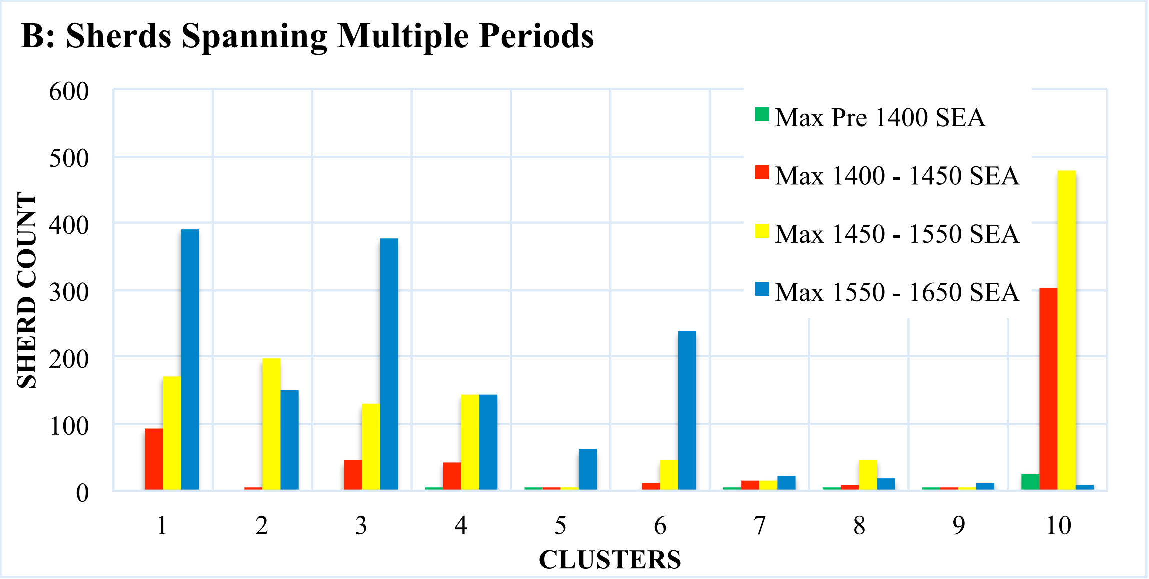

Fig. S4. Distribution of Southeast Asian trade ceramics by cluster, including all ceramics from Burma, Thailand and Vietnam. *A*. Only includes material definitely produced during one of the time periods. *B*. Counts of all material, including that which is not constrained within one of the time periods. These materials are counted in more than one period. Figure S4b thus gives a maximum limit for sherd counts for each period.

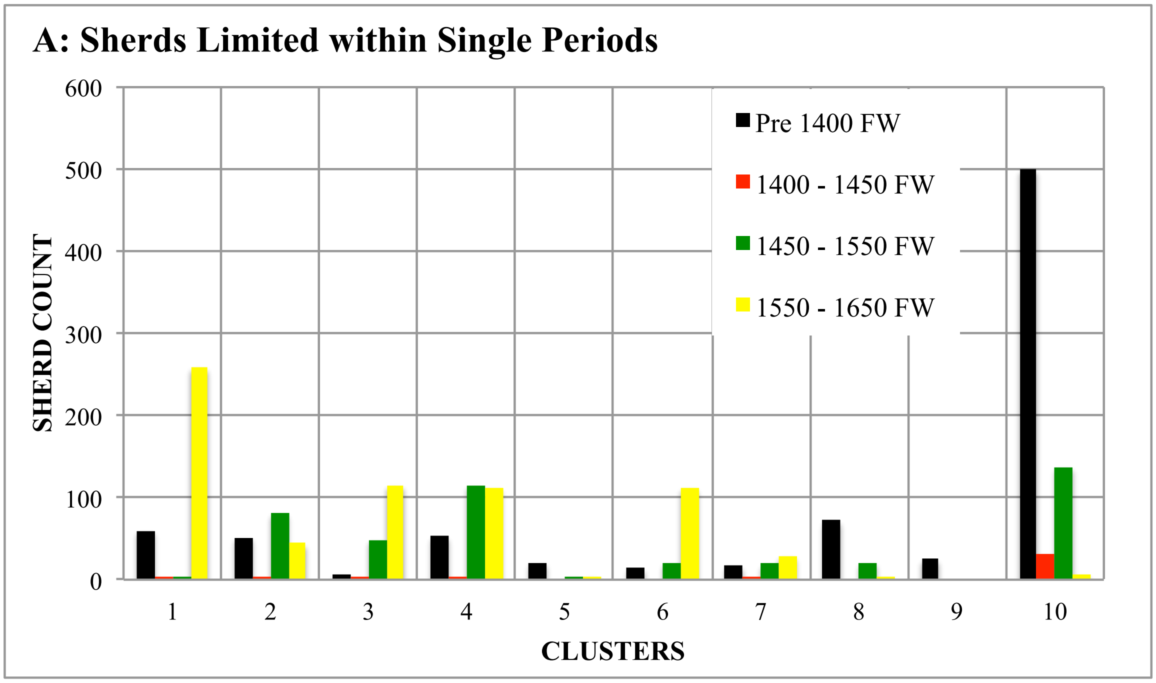

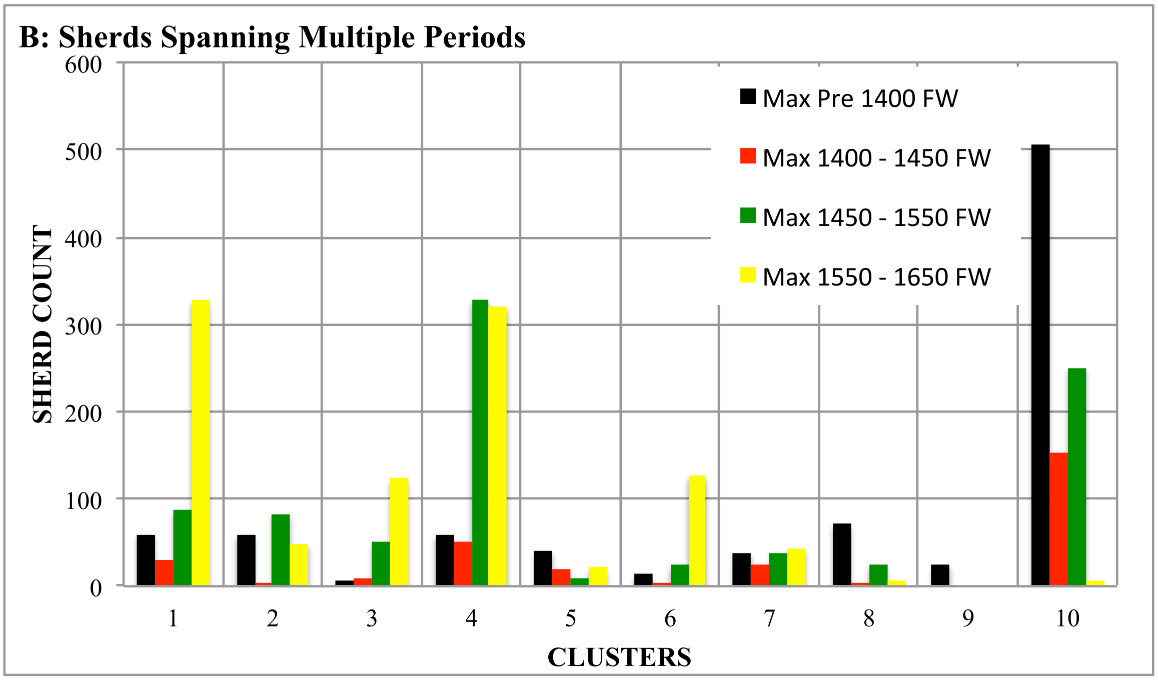

Fig. S5. Distribution Fine Ware (FW) ceramics by cluster. *A*. Includes only material that was certainly produced within one of the time periods. *B*. Counts of all material, including that which is not constrained within one of the time periods. These materials are counted in more than one period. Thus Figure S5B gives a maximum limit for sherd counts for each period.

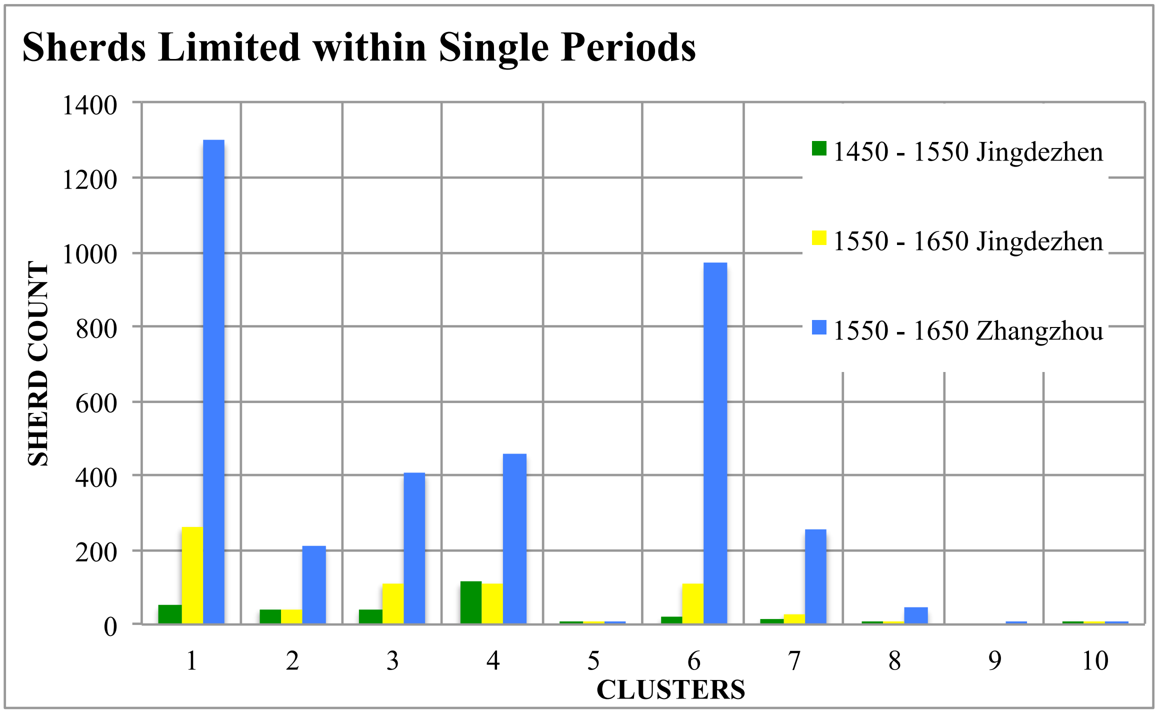

Fig. S6. Distribution of Chinese trade ceramics from the Jingdezhen and Zhangzhou production centers by cluster. Only includes material that fits within the time periods without overlap. Importantly, several types of distinctive and prominent Chinese trade ceramics that date after 1506 are barely present in Clusters 8 – 10, the former center for trade with China.

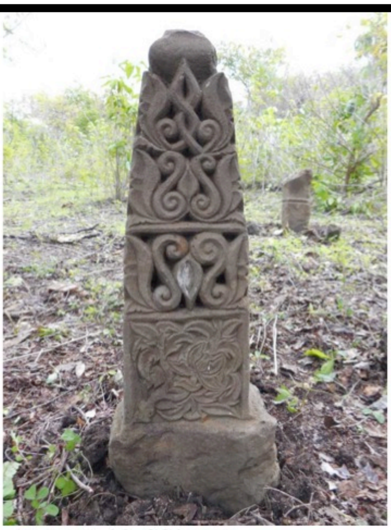

AB-LHR-30-GS3 N Face
16cm across 14cm thick 78cm tall
N: 05 36' 41.6"
E: 095 31' 54.3"

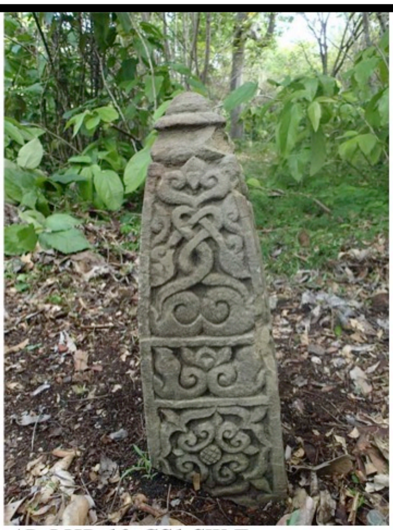

AB-LHR-10-GS1 SW Face
15cm across 11cm thick 46cm tall
N: 05 36' 39.6"
E: 095 31' 47.8"

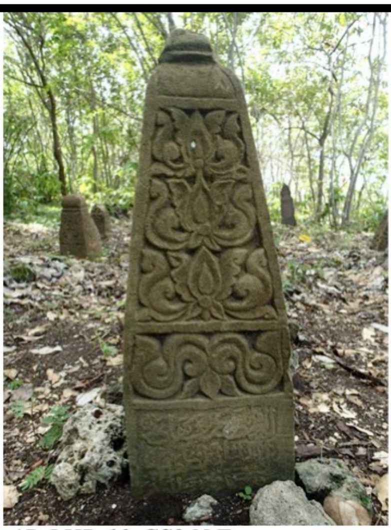

AB-LHR-10-GS8 N Face
19cm across 15cm thick 58cm tall
N: 05 36' 39.7"
E: 095 31' 47.7"

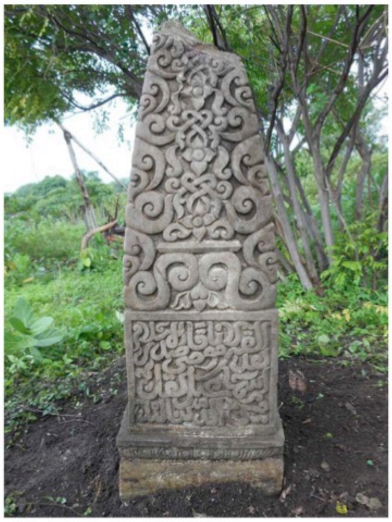

AB-LHR-50-GS1 N Face
27cm across 23cm thick 87cm tall
N: 05 36' 13.1"
E: 095 32' 01.2"

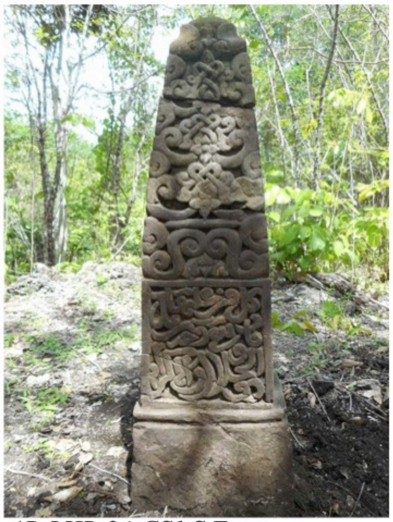

AB-LHR-24-GS1 S Face
24cm across 24cm thick 110cm tall
N: 05 36' 44.1"
E: 095 31' 51.0"

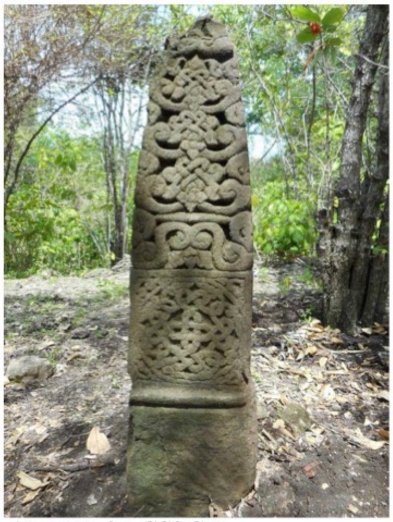

AB-LHR-24-GS2 S Face
24cm across 24cm thick 110cm tall
N: 05 36' 44.1"
E: 095 31' 51.1"

Fig. S7. Examples of *plang pleng* style gravestones recovered in our survey. All six of these are from the Lambaro headlands, that is within Cluster 10.

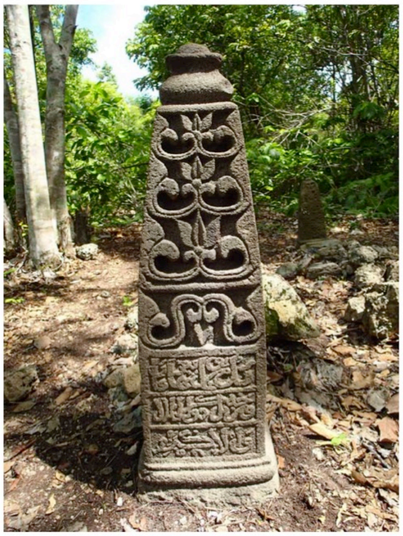

AB-LHR-10-GS13 S Face
19cm across 16cm thick 70cm tall
N: 05 36’ 39.6”
E: 095 31’ 47.6”

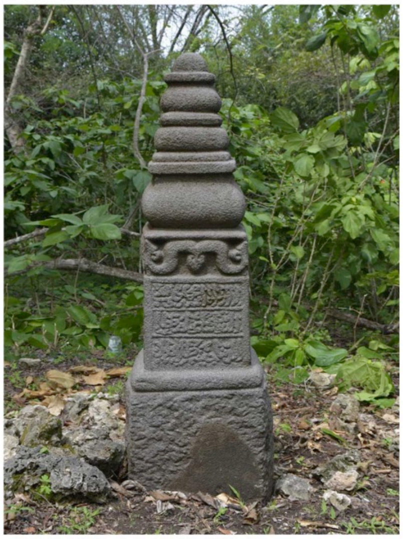

AB-LRH-44-GS1 SE Face
28cm across 23cm thick 122cm tall
N: 05 36’ 29.70”
E: 095 32’ 00.79”

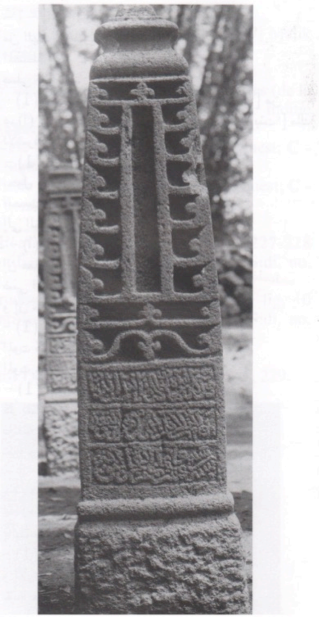

Kuta Kareung Cemetery
Tomb KK21 S. 1 & 2 Faces A
Date: 1415
Reference: Guillot & Kalus 2008

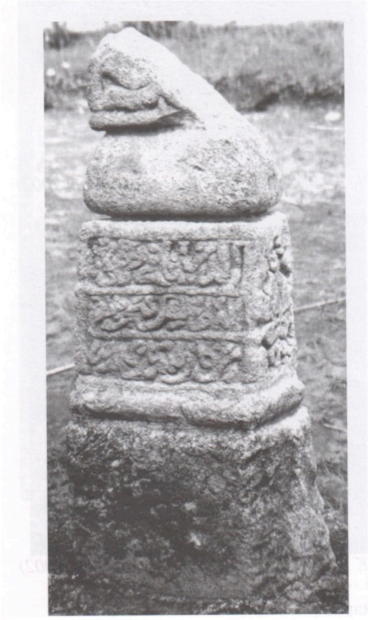

Tomb: TK I 05
Date: 1483-84
Reference: Guillot & Kalus 2008
Original Photo: Guillot & Kalus 2002

Fig. S8. Gravestones from our survey (top row) and 15th-century gravestones from Pasai (bottom row) are similar enough to imply that they date from the same period. Published examples from Guillot & Kalus 2008 (1).

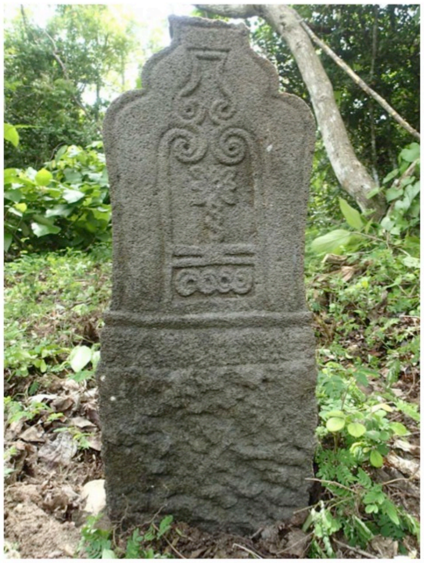

AB-LHR-41-GS1 NW Face
23cm across 8cm thick 63cm tall
N: 05 36' 30.5"
E: 095 31' 56.7"

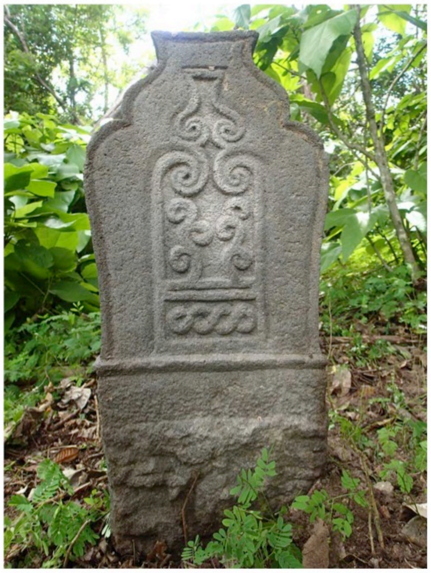

AB-LHR-41-GS2 N Face
24cm across 9cm thick 51cm tall
N: 05 36' 30.4"
E: 095 31' 56.7"

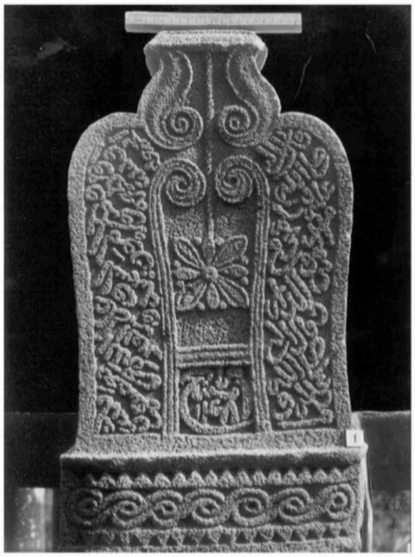

Teungku Sareh
Grave XIX (pair of stones)
Date: 1437
Reference: Lambourne 2004 p. 223-5

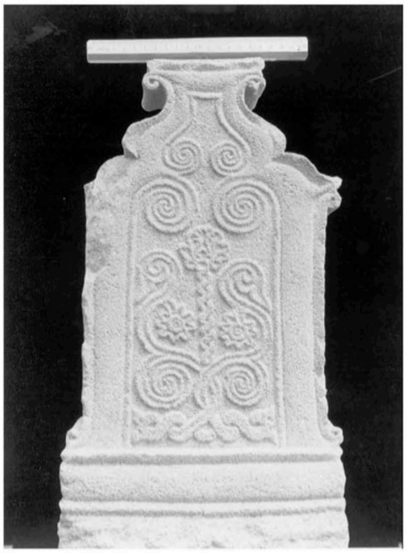

Peuet Ploh Peuet
Grave X (pair of stones)
Date: 1430
Reference: Lambourne 2004 p. 221-2

Fig. S9. Similarity in shape and decorative motifs between standing slab gravestones with curved sides from our survey (top row) and gravestones from Pasai dated to the 15th century (bottom row) imply that they date from the same period. Published examples from Lambourne 2004 (5).

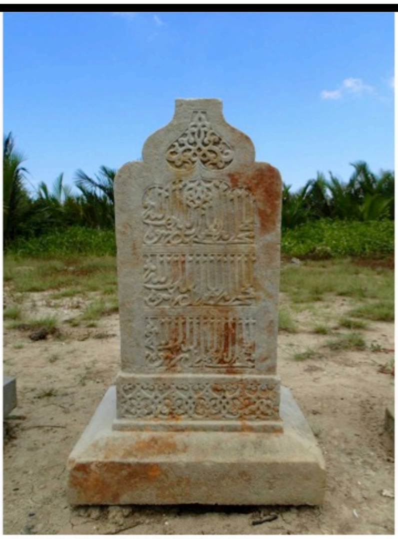

BA-PND-24-GS13 S Face
31cm across 20cm thick 79cm tall
N: 05 33’ 58.4”
E: 095 18’ 44.5”

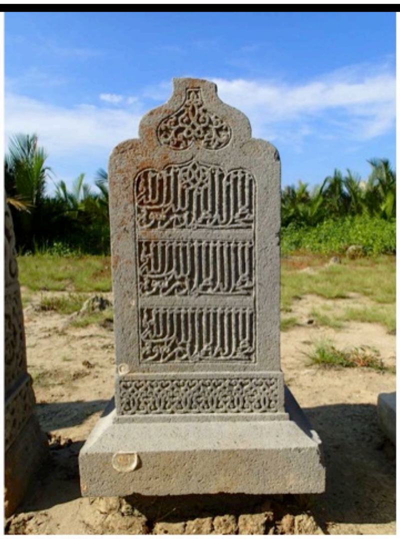

BA-PND-24-GS15 S Face
31cm across 21cm thick 83cm tall
N: 05 33’ 58.4”
E: 095 18’ 44.5”

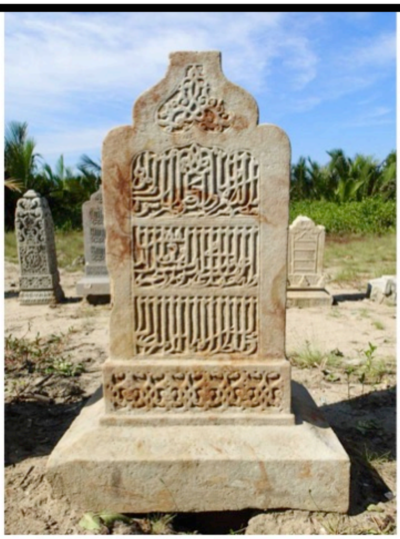

BA-PND-24-GS14 S Face
31cm across 20cm thick 75cm tall
N: 05 33’ 58.3”
E: 095 18’ 44.5”

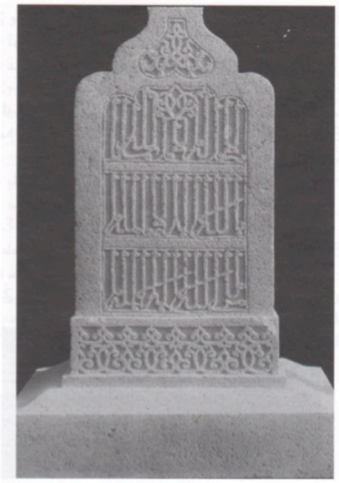

Tungku di Bale Cemetery
Tomb: TB 16
Date: 1517
Reference: Guillot & Kalus 2008

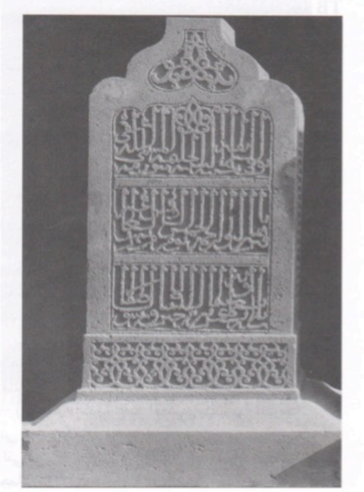

Tungku di Bale Cemetery
Tomb: TB 17
Date: 1517
Reference: Guillot & Kalus 2008

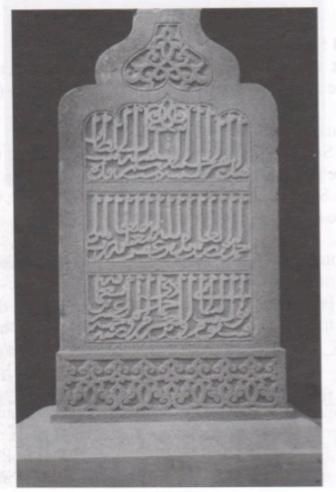

Tungku di Bale Cemetery
Tomb: TB 50
Date: 1519
Reference: Guillot & Kalus 2008

Fig. S10. Comparison of thick standing slab gravestones from our survey (top row) and gravestones from Pasai dated to the second decade of the 16th century. The shape and ornamentation are virtually identical. Our examples above come from the western part of our study area, within the city of Banda Aceh. Published examples from Guillot & Kalus 2008 (1).

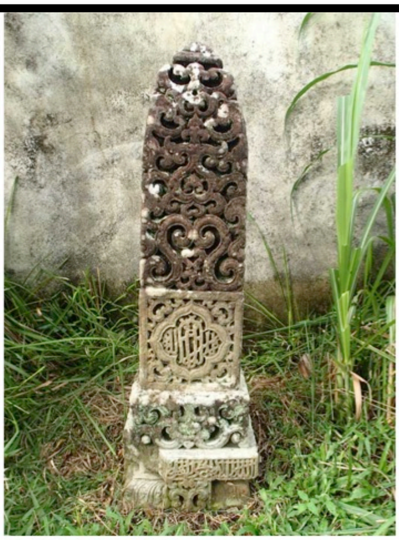

BA-LTH-1-GS10
29cm across 34cm thick 89cm tall
N: 05 32' 49.6"
E: 095 20' 40.1"

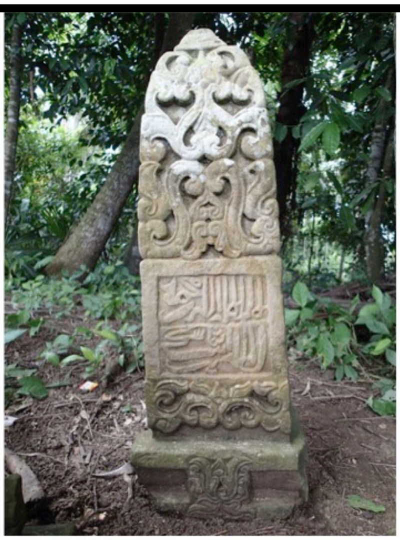

AB-LBT-1-GS1
23cm across 19cm thick 68cm tall
N: 05 30' 33.3"
E: 095 19' 59.1"

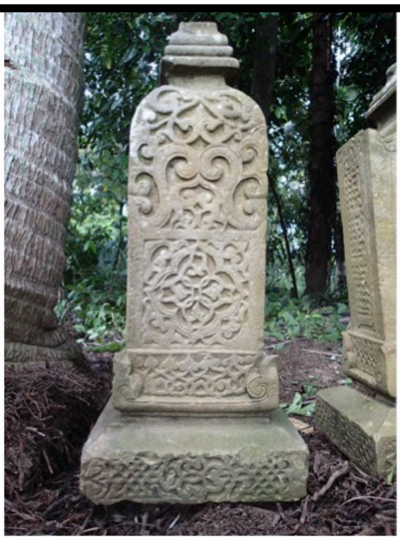

AB-LBT-1-GS3
27cm across 23cm thick 83cm tall
N: 05 30' 33.3"
E: 095 19' 59.2"

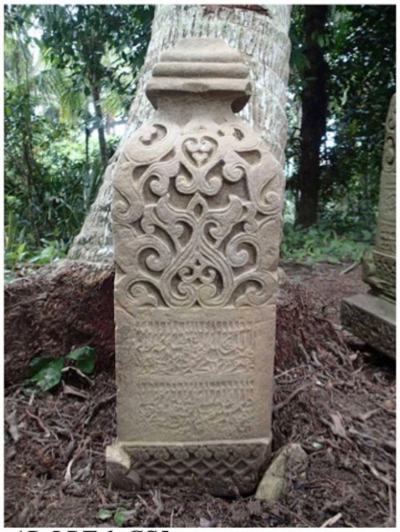

AB-LBT-1-GS5
21cm across 20cm thick 63cm tall
N: 05 30' 33.5"
E: 095 19' 59.1"

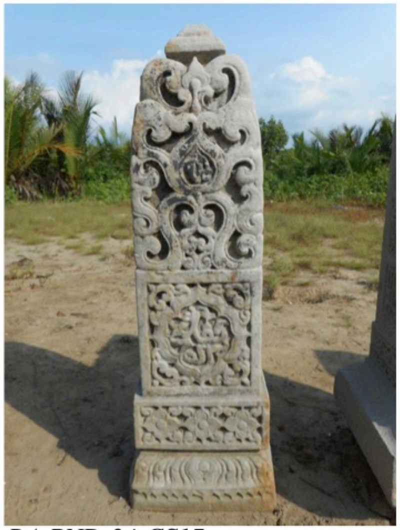

BA-PND-24-GS17
19cm across 19cm thick 80cm tall
N: 05 33' 58.4"
E: 095 18' 44.5"

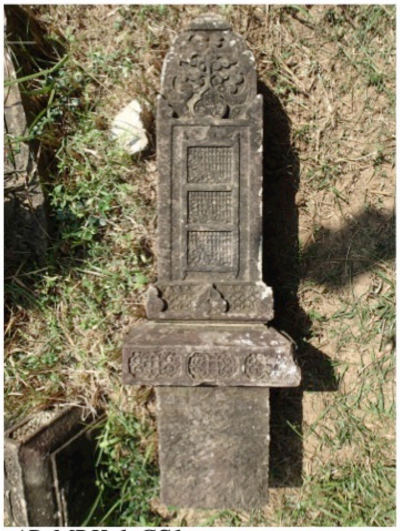

AB-MRK-1-GS1
27cm across 27cm thick 120cm tall
N: 05 32' 11.8"
E: 095 21' 21.5"

Fig. S11: Examples of what we believe are a post-15th century version of the *plang pleng* style of gravestones. These stones demonstrate more refined craftsmanship and incorporate elements, such as bases and finials, that are common elements of gravestone styles from the Aceh sultanate.

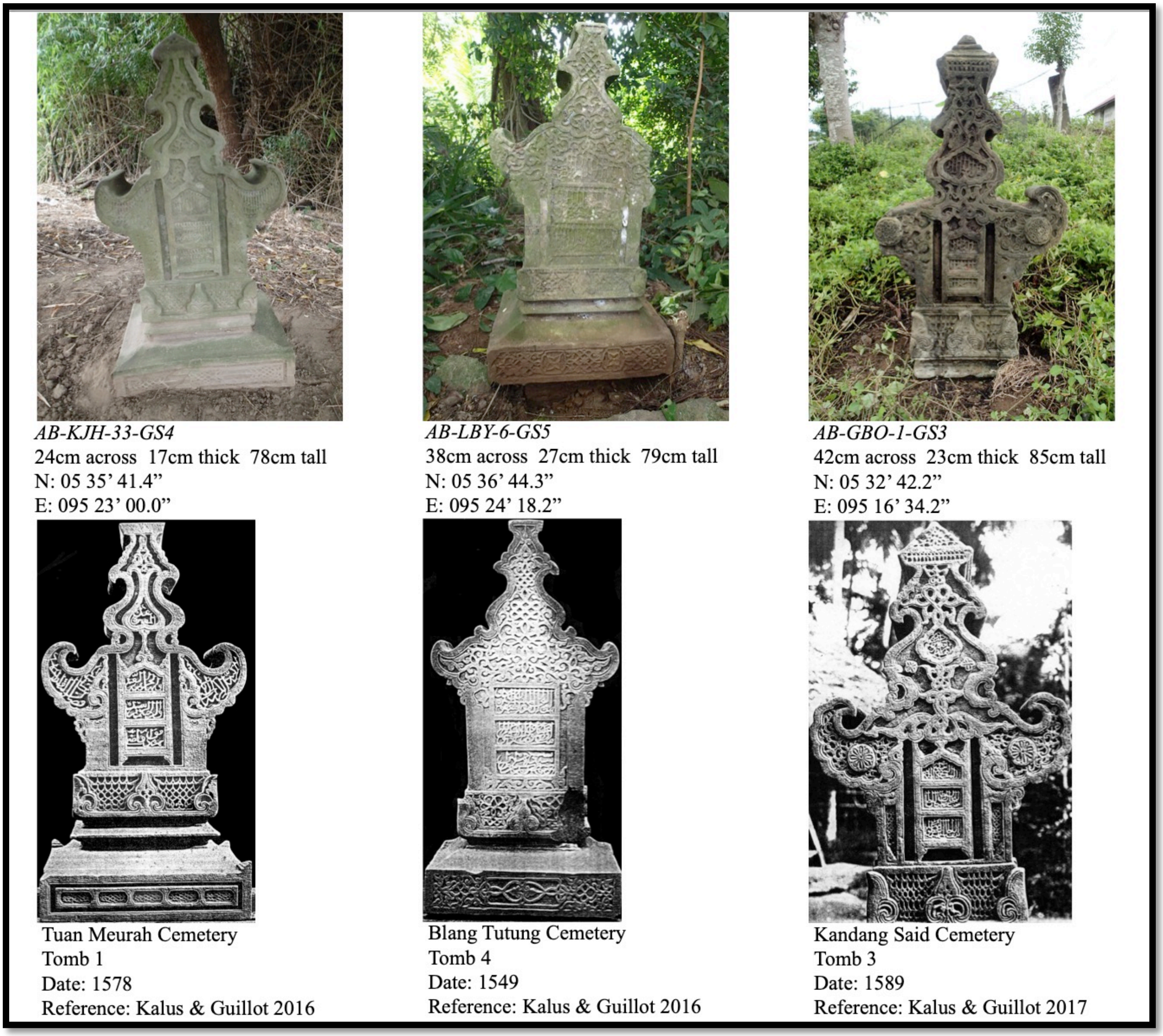

Fig. S12. Comparison of ‘winged’ gravestones from our survey (top row) and examples from the region that date from the late 16th century. Published examples from Kalus & Guillot 2016, 2017 (3 – 4).

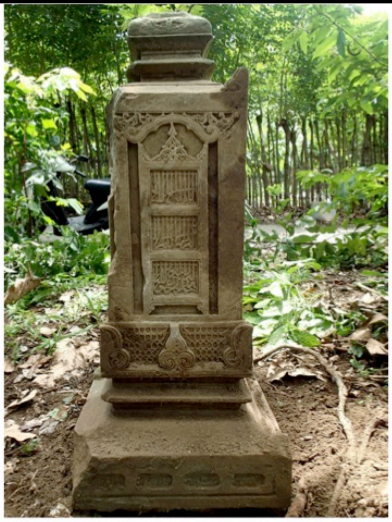

AB-LTT-2-GS9
29cm across 29cm thick 104cm tall
N: 05 32’ 35.3”
E: 095 15’ 17.1”

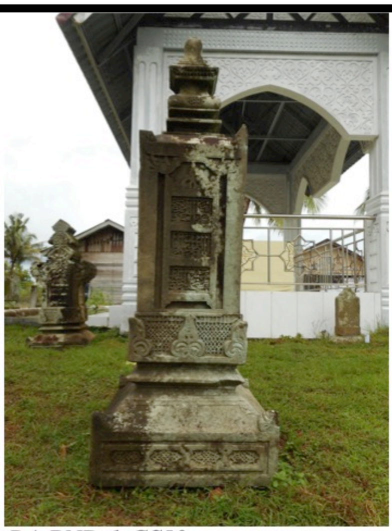

BA-PND-1-GS50
39cm across 39cm thick 171cm tall
N: 05 34’ 16.2”
E: 095 18’ 49.9”

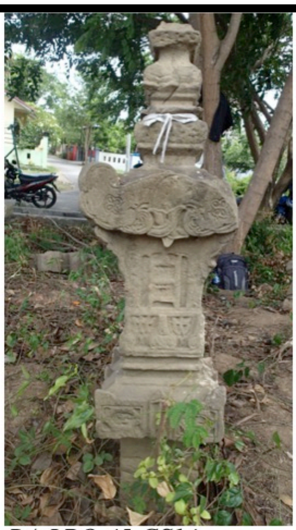

BA-LPO-45-GS14
32cm across 32cm thick 103cm tall
N: 05 34’ 35.6”
E: 095 19’ 28.6”

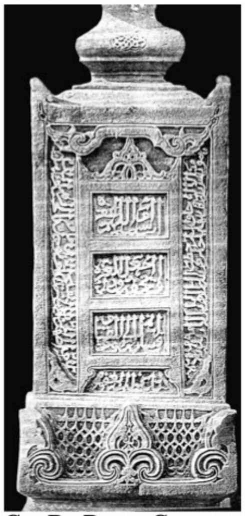

Cot Ba Beum Cemetery
Tomb 2
Date: 1579
Reference: Kalus & Guillot 2016

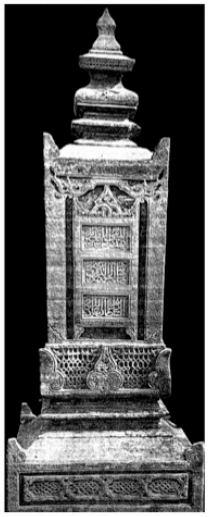

Tuan di Kandang Cemetery
Tomb 34
Date: 1589
Reference: Kalus & Guillot 2014

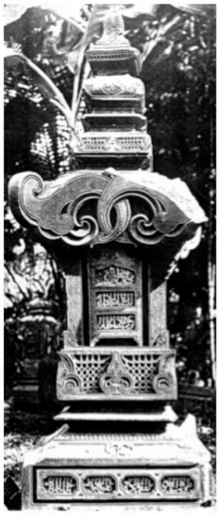

Kandang Kampung Punge Cemetery
Tomb 2
Date: 1593-94
Reference: Kalus & Guillot 2017

Fig. S13. Comparison of squared gravestones from our survey (top row) examples from the region dated to the late 16th century. Published examples from Kalus & Guillot 2014, 2016, & 2017 (2 – 4).

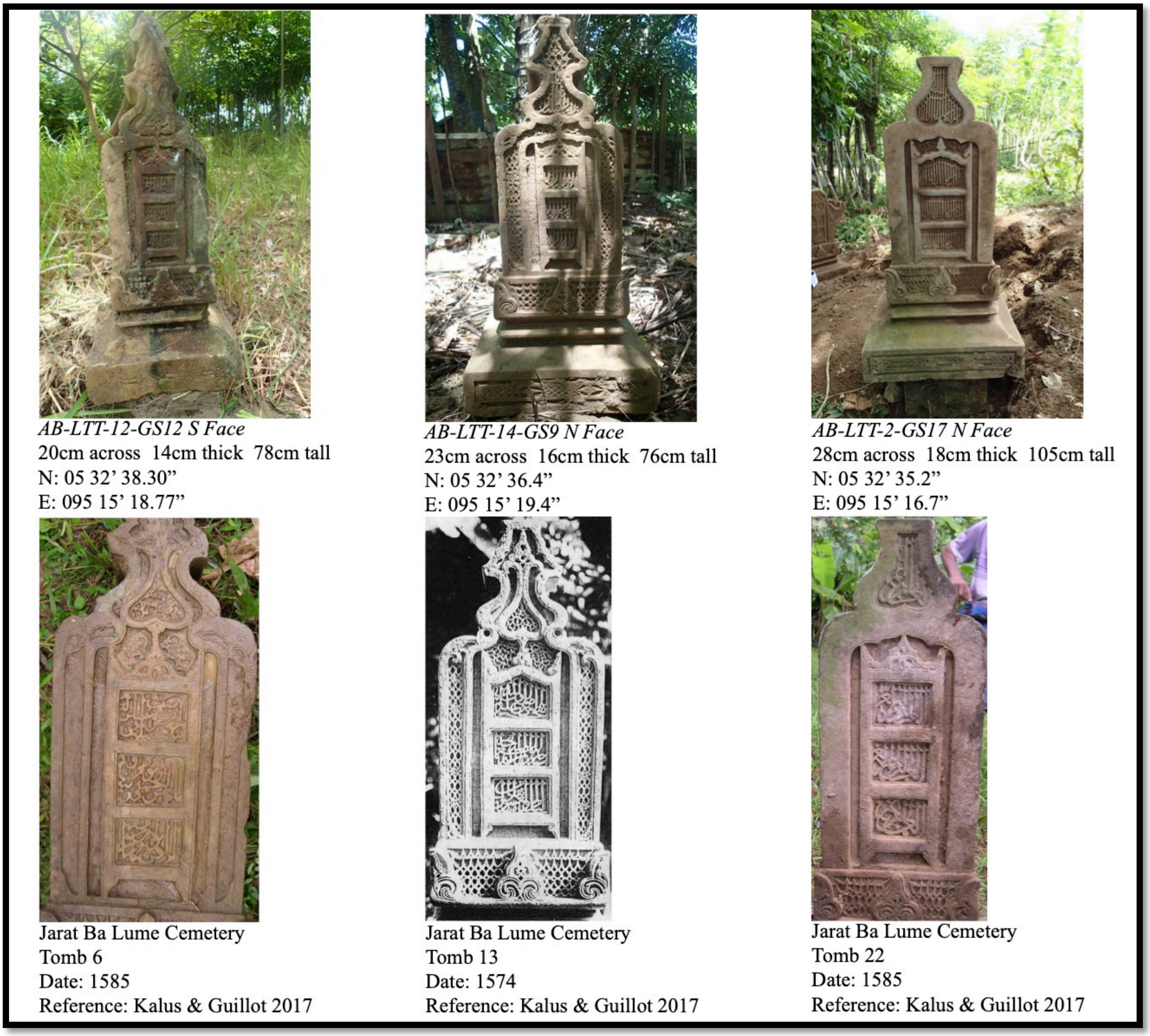

Fig. S14. Comparison of vertical slab gravestones with finials from our survey (top row) and examples from the region dated to the late 16th century. Published examples from Kalus & Guillot 2017 (4).

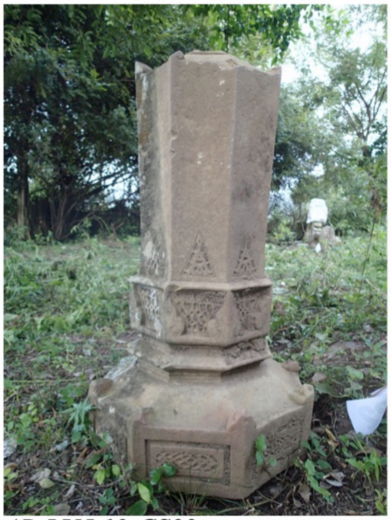

AB-LUJ-19-GS22
21cm dia. 86cm tall
N: 05 37' 16.2"
E: 095 24' 29.9"

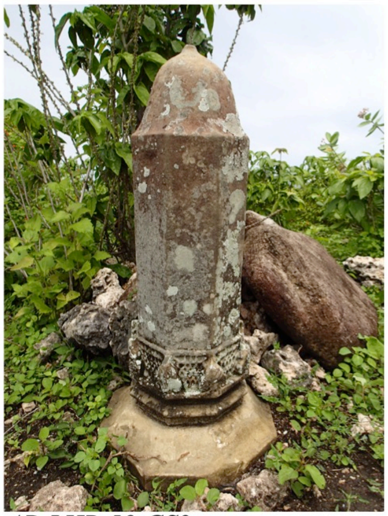

AB-LHR-59-GS2
23cm dia. 75cm tall
N: 05 36' 34.1"
E: 095 32' 05.4"

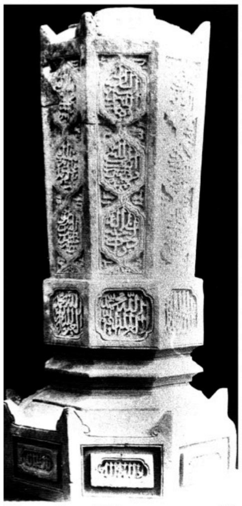

Tungku Glompang Cemetery
Tomb 1
Date: No 1587
Reference: Kalus & Guillot 2016

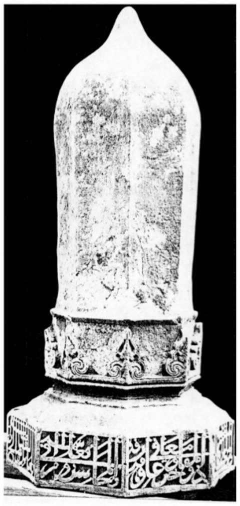

Tungku Syeh Aircam Cemetery
Tomb 1
Date: 1584
Reference: Kalus & Guillot 2017

Fig. S15. Comparison of hex and octagonal column gravestones from our survey (top row) with examples from the region dated to the late 16th century. Published examples from Kalus & Guillot 2016 & 2017 (3 – 4).

Supplementary Tables

Table S1. Quantity of ceramics sherds dated with confidence by period for each cluster. Showing total count of entire assemblage, count of all sherds of 'high class' fine wares, and count of sherds of large jars.

	Total Count					Fine Ware					Large Jars			
Period	Pre 1400	1400 - 1450	1450 - 1550	1550 - 1650	1650 – 1800	Pre 1400	1400 - 1450	1450 - 1550	1550 - 1650	1650 - 1800	Pre 1400	1400 - 1450	1400 - 1550	1550 - 1750
Cluster 1	216	2	84	1613	2737	60	2	4	258	861	123	0	27	222
Cluster 2	165	6	240	453	774	52	2	81	44	228	38	0	0	34
Cluster 3	69	1	107	591	727	7	1	49	115	304	49	0	23	250
Cluster 4	70	0	368	594	1740	53	2	115	112	486	7	0	0	312
Cluster 5	23	0	5	144	631	21	0	5	5	81	0	0	0	55
Cluster 6	54	0	45	1157	2084	14	0	21	112	452	44	0	10	192
Cluster 7	148	2	23	572	2426	17	2	19	29	337	8	2	0	283
Cluster 8	412	1	41	48	90	72	0	21	4	9	266	1	5	10
Cluster 9	151	0	2	8	89	25	0	0	0	5	46	0	1	12
Cluster 10	985	60	210	6	0	499	31	137	6	0	1092	6	106	8
Total	2293	72	1125	5186	11298	820	40	452	685	2763	1673	9	172	1378

Table S2. Quantity of diagnostic Chinese trade ceramics, dated with confidence by period for each cluster. Showing total count of material from production centers in the Longquan, Zingdezhen, and Zhangzhou provinces.

	Longquan		Jingdezhen		Zhangzhou
Period	Pre 1400	1400 - 1450	1450 – 1550	1550 1650	1550 - 1650
Cluster 1	60	2	51	262	1298
Cluster 2	50	2	43	42	211
Cluster 3	5	1	41	112	407
Cluster 4	48	0	113	112	460
Cluster 5	21	0	5	6	1
Cluster 6	14	0	19	111	969
Cluster 7	22	2	17	29	254
Cluster 8	74	0	2	4	44
Cluster 9	25	0	0	0	8
Cluster 10	417	17	10	6	1
Total	736	24	301	684	3653

Table S3. Quantity of ceramics sherds dated with confidence by period for each cluster from Burmese, Thai, Vietnamese, and Japanese production centers.

	Total Count Burma					Total Count Thailand					Total Count Vietnam					Total Count Japan				
Period	Pre 1400	1400 – 1450	1450 - 1550	1550 - 1650	1650 – 1800	Pre 1400	1400 – 1450	1450 1550	1550 - 1650	1650 – 1800	Pre 1400	1400 - 1450	1450 - 1550	1550 - 1650	1650 - 1800	Pre 1400	1400 - 1450	1450 - 1550	1550 - 1650	16 - 18
Cluster 1	0	0	31	108	0	0	0	1	2	0	0	0	0	0	0	0	0	0	2	62
Cluster 2	0	1	157	82	4	0	1	38	0	0	0	0	0	0	13	0	0	0	2	17
Cluster 3	0	0	61	68	8	0	0	5	0	0	0	0	0	0	38	0	0	0	2	18
Cluster 4	0	0	0	1	350	0	0	46	0	0	0	0	8	0	47	0	0	0	0	33
Cluster 5	0	0	0	0	4	1	0	4	0	0	0	0	0	0	0	0	0	0	0	57
Cluster 6	0	0	17	66	31	0	0	9	0	0	0	0	0	0	202	0	0	0	1	33
Cluster 7	0	0	1	13	309	0	0	1	0	0	0	0	1	0	130	0	0	0	0	31
Cluster 8	0	0	19	0	0	0	0	19	0	0	0	0	1	0	0	0	0	0	0	8
Cluster 9	0	0	2	0	0	1	0	0	0	0	0	0	0	0	4	0	0	0	0	2
Cluster 10	4	10	72	0	0	4	18	127	0	0	9	7	0	0	0	0	0	0	0	0
Total	4	11	360	338	706	6	19	250	2	0	9	7	10	0	434	0	0	0	7	20

Table S4. Counts of gravestones from the survey by time period. The post-mid 16th century graves are mostly types known to be associated with the Aceh sultanate.

**Period (C.E.)**	**Count**
15th Century	152
Late 15th to Mid 16th Century	116
Post Mid 16th Century	1598
Undated	3997
Total	5863

Table S5. Reference set of dated gravestones from the region.

Example	Dates	References
**15th Century Gravestone Types**		
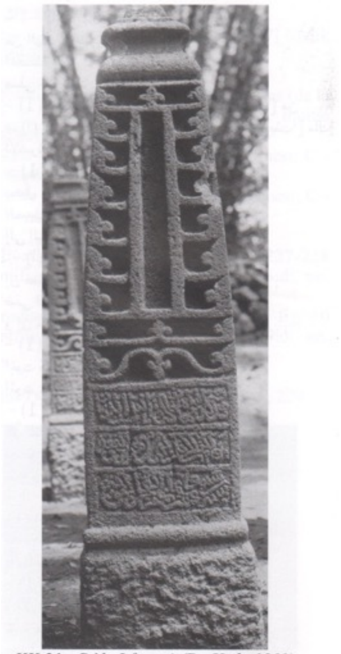 *KK 21 : Stèle I face A (De Vink, 1911)*	1415	Kuta Kareung Cemetery Tomb KK21 (1)
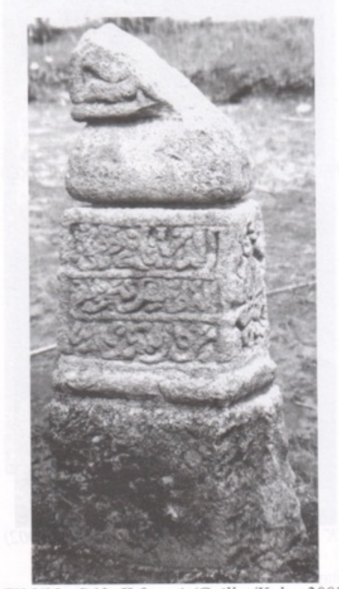 *TK I/05 : Stèle II face A (Guillot/Kalus 2002)*	1483 - 1484	Tomb TK I 05 (1)

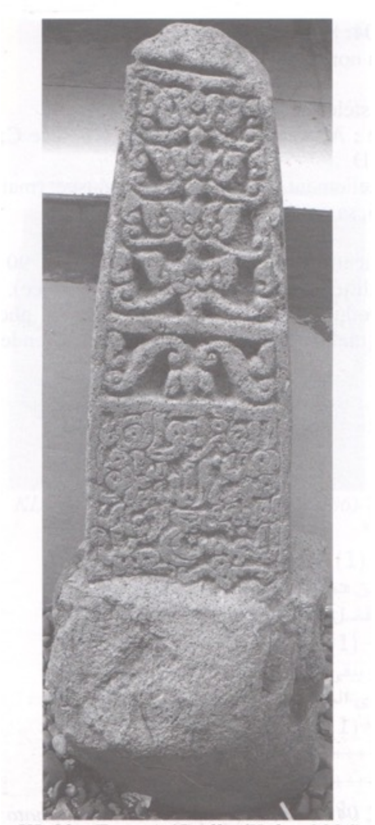 KL 02 : Face A (Guillot/Kalus 2006)	1406 - 1407	AB Lamreh Kota Lubhok Cemetery Tomb KL01 (1)
	1421	AB Lamreh Kota Lubhok Cemetery Tomb KL03 (1)
	1437	AB Lamreh Kota Lubhok Cemetery Tomb KL04 (1)
	1441	Tomb TK II 17 (1)
	1441	AB Lamreh Kota Lubhok Cemetery Tomb KL02 (1)
	1446	Tomb TK I 03 (1)
	1457 - 1458	Ab Glee Jong Cemetery Tomb GJ04 (1)
	1458	Tomb TK I 07 (1)
	1460	Tomb YK I 04 (1)
	1461	Tomb TK I 19 (1)
	1471	Ab Glee Jong Cemetery Tomb GJ01 (1)
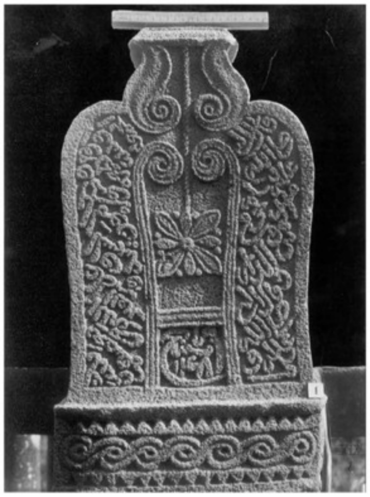	1430	Peuet Ploh Peuet Grave X (5)
	1435	Peut Pleug Peut Cemetery (1)
	1436	Tungku Sareh Cemetery Tomb TSA 19 (1)
	1437	Teungku Sareh Grave XIX (5)
	1437 - 1438	Teungku Sidi Grave VII (5)
	1438	Kuta Kareung Cemetery Tomb KK20 (1)
	1440	Tungku Sareh Cemetery Tomb TSA 18 (1)

Late 15th – Early 16th Century Gravestone Types

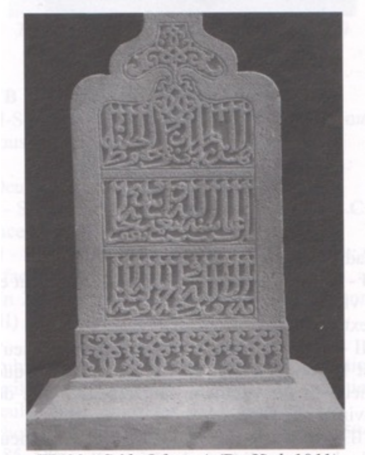

TB 09 : Stèle I face A (De Vink 1911)

1431	Tungku di Bale Cemetery Tomb TB41 (1)
1467 - 1468	Tungku di Bale Cemetery Tomb TB10 (1)
1468	Tungku di Bale Cemetery Tomb TB58 (1)
1472	Kuta Kareung Cemetery Tomb KK16 (1)
1473 - 1474	Kuta Kareung Cemetery Tomb KK01 (1)
1475	Tungku di Bale Cemetery Tomb TB03 (1)
1475	Tungku di Bale Cemetery Tomb TB38 (1)
1476	Tungku di Bale Cemetery Tomb TB43 (1)
1477	Tungku di Bale Cemetery Tomb TB46 (1)
1480	Tungku di Bale Cemetery Tomb TB62 (1)
1484	Tungku di Bale Cemetery Tomb TB47 (1)
1485	Tungku di Bale Cemetery Tomb TB07 (1)
1485 (or 1466)	Tungku di Bale Cemetery Tomb TB39 (1)
1489 (or 1470)	Tungku di Bale Cemetery Tomb TB35 (1)
1490	Tungku di Bale Cemetery Tomb TB02 (1)
1490	Tungku di Bale Cemetery Tomb TB48 (1)
1492	Tungku di Bale Cemetery Tomb TB08 (1)
1492	Tungku di Bale Cemetery Tomb TB34 (1)
1494 - 1495	Tungku di Bale Cemetery Tomb TB36 (1)
1495	Tungku di Bale Cemetery Tomb TB33 (1)
1495	Tungku di Bale Cemetery Tomb TB37 (1)
1499	Peut Pleug Peut Cemetery Tomb PP01bis (1)
1500	Tungku Sareh Cemetery Tomb TSA07 (1)
1502	Peut Pleug Peut Cemetery Tomb PP01 (1)
1506	Tomb TM02 (1)
1507	Tungku di Bale Cemetery Tomb TB09 (1)
1508	Tungku Sareh Cemetery Tomb TSA10 (1)
1509	Tungku di Bale Cemetery Tomb TB14 (1)
1509	Tungku di Bale Cemetery Tomb TB15 (1)
1510	Tomb TM03 (1)
1510	Tungku Sareh Cemetery Tomb TSA22 (1)
1511	Tungku di Bale Cemetery Tomb TB28 (1)
1513	Tungku di Bale Cemetery Tomb TB21 (1)
1513	Tungku di Bale Cemetery Tomb TB22 (1)
1514	Tungku di Bale Cemetery Tomb TB19 (1)

	1514	Tungku di Bale Cemetery Tomb TB01 (1)
	1514	Tungku Sareh Cemetery Tomb TSA11 (1)
	1517	Tungku di Bale Cemetery Tomb TB16 (1)
	1517	Tungku di Bale Cemetery Tomb TB17 (1)
	1517	Tungku di Bale Cemetery Tomb TB51 (1)
	1517	Tungku Sareh Cemetery Tomb TSA13 (1)
	1519 (or 1509)	Tungku di Bale Cemetery Tomb TB23 (1)
	1519	Tungku di Bale Cemetery Tomb TB50 (1)
**Mid 16th Century – 18th Century Gravestone Types**		
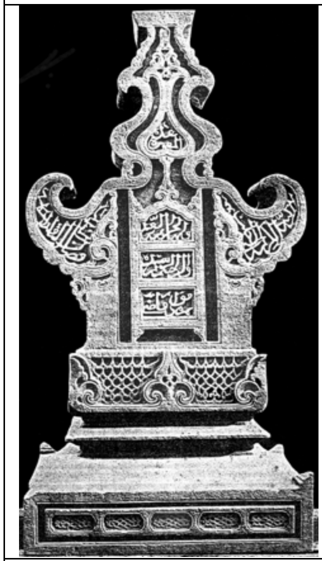	1549	Blang Tutung Cemetery Tomb 4 (3)
	1563	Meuseudjit Raya-Raya Cemetery Tomb 2 (4)
	1565	Yub Hagu Cemetery (4)
	1573 - 1582	Tuan di Kadang Cemetery Tomb 35 (2)
	1578	Tuan Meurah Cemetery Tomb 1 (3)
	1579	Kandang Kampung Punge Cemetery Tomb 1 (4)
	1589	Kandang Said Cemetery Tomb 3 (4)
	1591	Tuan di Kandang Cemetery Tomb 5 (4)
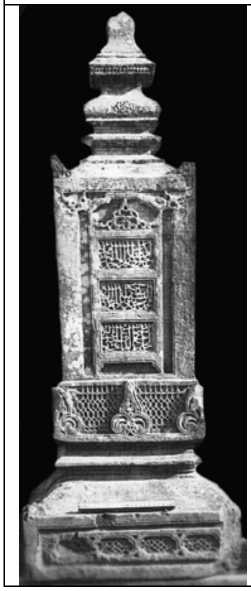	1579	Cot Ba Beum Cemetery Tomb 2 (3)
	1580 - 1581	Tuan di Guri Cemetery Tomb 5 (4)
	1585	Tungku Ba Asan Cemetery Tomb 14 (3)
	1586 - 1587	Jarat Raya Cemetery Tomb 8 (3)
	1589	Tuan di Kandang Cemetery Tomb 34 (2)
	1591	Tungku Lam Raya Cemetery Tomb 1 (4)
	1591	Tuan di Kandang Cemetery III Tomb 6 (2)
	1594	Tuan Meurah Cemetery Tomb 2 (3)

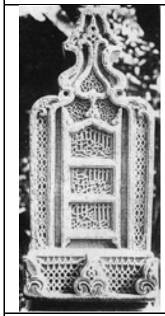	1573 - 1574	Jarat Ba Lume Cemetery Tomb 1 (4)
	1574	Jarat Ba Lume Cemetery Tomb 13 (4)
	1585	Jarat Ba Lume Cemetery Tomb 6 (4)
	1585	Jarat Ba Lume Cemetery Tomb 22 (4)
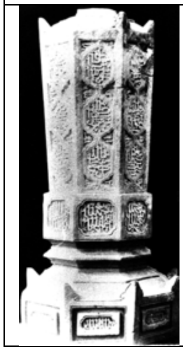	1584	Tungku Syeh Aircam Cemetery Tomb 1 (4)
	1587	Tungku Glompang Cemetery Tomb 1 (3)

Dataset S1 (separate file)

Ceramic counts by Cluster and time period used to produce the GIS distribution maps used in this paper.

References

1. Guillot, C. & Kalus, L. (2008). *Les monuments funéraires et l'histoire du Sultanat du Pasai*. Paris: Cahiers d'Archipel.
2. Kalus, L. & Guillot, G. 2014. Cimetière de Tuan di Kandang*, Archipel* 88: 71-147.
3. Kalus, L. & Guillot, G. 2016. Cimetières de Sumatra, Varia. I [Épigraphie islamique d'Aceh. 10]. *Archipel* 91 : 55-103.
4. Kalus, L. & Guillot, G. 2017. Cimetières de Sumatra, Varia. II [Épigraphie islamique d'Aceh. 11] *Archipel* 93 : 31-84.
5. Lambourn, E. 2004. The formation of the batu Aceh tradition in fifteenth-century Samudera-Pasai. *Indonesia and the Malay World*, 32: 211-248.
6. Perret, D., Razak, K. & Kalus, L. 1999. *Batu Aceh: Warisan sejarah Johor*. Johor Bahru: Yayasan Warisan Johor/École Française d'Extrême-Orient.
7. Perret, D., Razak, K. & Kalus, L. 2004. *Batu Aceh Johor Dalam Perbandingan*. Johor Bahru: Yayasan Warisan Johor/ École Française d'Extrême-Orient.
8. Haslett, J., & Parnell, A. C. (2008). A simple monotone process with application to radiocarbon-dated depth chronologies. *Journal of the Royal Statistical Society: Series C (Applied Statistics)*, *57*(4), 399–418. https://doi.org/10.1111/j.1467-9876.2008.00623.x
9. Parnell, A. C., Haslett, J., Allen, J. R. M., Buck, C. E., & Huntley, B. (2008). A flexible approach to assessing synchroneity of past events using Bayesian reconstructions of sedimentation history. *Quaternary Science Reviews*, *27*(19–20), 1872–1885. https://doi.org/10.1016/j.quascirev.2008.07.009
10. Fraley, C., & Raftery, A. E. (2002). Model-based clustering, discriminant analysis, and density estimation. *Journal of the American Statistical Association*, *97*(458), 611–631. https://doi.org/10.1198/016214502760047131
11. Scrucca L., Fop M., Murphy T. B. and Raftery A. E. (2017) mclust 5: clustering, classification and density estimation using Gaussian finite mixture models. *The R Journal* 8/1, pp 205-233
12. Bronk Ramsey, C. (1995). Radiocarbon calibration and analysis of stratigraphy: The OxCal program. *Radiocarbon*, 37(2), 425 – 430.
13. Meltzner, A. J. *et al.* (2010). Coral evidence for earthquake recurrence and an A.D. 1390-1455 cluster at the south end of the 2004 Aceh-Andaman rupture. *Journal of Geophysical Research-Solid Earth* **115**, doi:B10402 10.1029/2010jb007499
14. Sieh. K. *et al.* (2015). Penultimate predecessors of the 2004 Indian Ocean tsunami in Aceh, Sumatra: Stratigraphic, archaeological, and historical evidence. *Journal of Geophysical Research-Solid Earth* 120, 308 – 325; doi:10.1002/2014jb011538
