# Supplementary material for: Archaeological evidence that a late 14th-century tsunami devastated the coast of northern Sumatra and redirected history
Source: Proc Natl Acad Sci U S A. 2019 May 28;116(24):11679–86. doi: 10.1073/pnas.1902241116 (PMC6587673; doi:10.1073/pnas.1902241116)
Supplement: Supplementary File [file pnas.1902241116.sd01.pdf]

CLUSTER 1 TRADE CERAMICS

Country	Region	Sub-Region	Basin	Bowl	Dish	Jar	Lid	Washer
China	Fujian	Cizao				7		
China	Fujian	Tongan		49				
China	Fujian	Tongan			5			
China	Guangdong		10					
China	Guangdong					123		
China	Guangdong						2	
China	Zhejiang	Longquan		30				
China	Zhejiang	Longquan			27			
China	Zhejiang	Longquan						3

Cluster 1 Pre-1400 All. We recovered 256 sherds from Cluster 1 that date prior to 1400. There are no overlapping sherds for this period.

Country	Region	Sub-Region	Bowl	Dish
China	Zhejiang	Longquan	1	
China	Zhejiang	Longquan		1

Cluster 1 1400 – 1450 Definite. We recovered 2 sherds that were produced between 1400 and 1450.

Country	Region	Sub-Region	Basin	Bowl	Covered Box	Dish	Jar	Kendi	Lid
Burma	Mon	Martaban	1						
Burma	Mon	Martaban		3					
Burma	Mon	Martaban				31			
Burma	Mon	Martaban					22		

China	Zhejiang	Longquan		1					
China	Zhejiang	Longquan				1			
Thailand	Sukhothai	Sawankhalok		2					
Thailand	Sukhothai	Sawankhalok				26			
Thailand	Sukhothai	Sukhothai			1				
Thailand	Sukhothai	Sukhothai				2			
Thailand	Sukhothai	Sukhothai							2
Thailand						1			
Vietnam	Hai Duong					1			
Vietnam	Hai Duong							1	

Cluster 1 1400 – 1450 Possible. We recovered 95 sherds that might have been produced between 1400 and 1450.

Country	Region	Sub-Region	Bowl	Covered Box	Dish	Jar	Jarlet	Unknown
Burma	Mon	Martaban	12					
Burma	Mon	Martaban			15			
Burma	Mon	Martaban				4		
China	Guangdong					1		
China	Jiangxi	Jingdezhen	10					
China	Jiangxi	Jingdezhen			38			
China	Jiangxi	Jingdezhen				1		
China	Jiangxi	Jingdezhen					1	
China	Jiangxi	Jingdezhen						1
Thailand	Sukhothai	Sawankhalok		1				

Cluster 1 1450 – 1550 Definite. We recovered 84 sherds that were produced between 1450 and 1550.

Country	Region	Sub-Region	Basin	Bowl	Covered Box	Dish	Jar	Jarlet	Kendi	Lid	Unknown
Burma	Mon	Martaban	1								
Burma	Mon	Martaban		20							
Burma	Mon	Martaban				76					
Burma	Mon	Martaban					31				
China	Guangdong						1				
China	Jiangxi	Jingdezhen		10							
China	Jiangxi	Jingdezhen				38					
China	Jiangxi	Jingdezhen					1				
China	Jiangxi	Jingdezhen						1			
China	Jiangxi	Jingdezhen									1
Thailand	Sukhothai	Sawankhalok		2							
Thailand	Sukhothai	Sawankhalok			1						
Thailand	Sukhothai	Sawankhalok				30					
Thailand	Sukhothai	Sawankhalok					3				
Thailand	Sukhothai	Sukhothai			1						
Thailand	Sukhothai	Sukhothai				2					
Thailand	Sukhothai	Sukhothai								2	
Thailand						1					
Vietnam	Hai Duong					1					
Vietnam	Hai Duong								1		

Cluster 1 1450 – 1550 Possible. We recovered 224 sherds that might have been produced between 1450 and 1550.

Country	Region	Sub-Region	Bowl	Covered Box	Cup	Dish	Jar	Jarlet	Lid	Potiche	Stem Cup	Unknown
Burma	Mon	Martaban	7									
Burma	Mon	Martaban				19						
Burma	Mon	Martaban					80					
Burma	Mon	Martaban							1			
Burma	Mon	Martaban										1
China	Fujian	Dehua	2									
China	Fujian	Dehua								1		
China	Fujian	Zhangzhou	214									
China	Fujian	Zhangzhou		2								
China	Fujian	Zhangzhou				1014						
China	Fujian	Zhangzhou							3			
China	Fujian	Zhangzhou								8		
China	Fujian	Zhangzhou										1
China	Guangdong						2					
China	Jiangxi	Jingdezhen	140									
China	Jiangxi	Jingdezhen		8								
China	Jiangxi	Jingdezhen			5							
China	Jiangxi	Jingdezhen				81						
China	Jiangxi	Jingdezhen					1					
China	Jiangxi	Jingdezhen						1				
China	Jiangxi	Jingdezhen							1			
China	Jiangxi	Jingdezhen								8		
China	Jiangxi	Jingdezhen									2	
China	Jiangxi	Jingdezhen										1
Japan	Hizen					2						

Thailand	Sukhothai	Sawankhalok					2					

Cluster 1 1550 – 1650 Definite. We recovered 1,607 sherds that were produced between 1550 and 1650.

Country	Region	Sub-Region	Bowl	Covered Box	Cup	Dish	Jar	Jarlet	Lid	Potiche	Stem Cup	Unknown
Burma	Mon	Martaban	29									
Burma	Mon	Martaban				127						
Burma	Mon	Martaban					225					
Burma	Mon	Martaban							1			
Burma	Mon	Martaban										1
China	Fujian	Dehua	4									
China	Fujian	Dehua		8								
China	Fujian	Dehua			1							
China	Fujian	Dehua				1						
China	Fujian	Dehua							20			
China	Fujian	Dehua								29		
China	Fujian	Zhangzhou	256									
China	Fujian	Zhangzhou		2								
China	Fujian	Zhangzhou				1025						
China	Fujian	Zhangzhou							5			
China	Fujian	Zhangzhou								9		
China	Fujian	Zhangzhou										1
China	Guangdong						2					
China	Jiangxi	Jingdezhen	143									
China	Jiangxi	Jingdezhen		8								
China	Jiangxi	Jingdezhen			5							

China	Jiangxi	Jingdezhen				85						
China	Jiangxi	Jingdezhen					1					
China	Jiangxi	Jingdezhen						1				
China	Jiangxi	Jingdezhen							7			
China	Jiangxi	Jingdezhen								9		
China	Jiangxi	Jingdezhen									2	
China	Jiangxi	Jingdezhen										1
Japan	Hizen					2						
Thailand	Sukhothai	Sawankhalok			4							
Thailand	Sukhothai	Sawankhalok				5						

Cluster 1 1550 – 1650 Possible. We recovered 2,019 sherds that might have been produced between 1550 and 1650.

Country	Region	Sub-Region	Bowl	Covered Box	Cup	Dish	Jar	Kendi	Lid	Potiche	Vase	Unknown
China	Fujian	Anxi	3									
China	Fujian	Dehua	138									
China	Fujian	Dehua				25						
China	Fujian	Dehua							1			
China	Fujian	Dehua								2		
China	Fujian	Zhangzhou	1341									
China	Fujian	Zhangzhou		1								
China	Fujian	Zhangzhou			1							
China	Fujian	Zhangzhou				151						
China	Fujian	Zhangzhou					8					
China	Fujian	Zhangzhou							10			
China	Fujian	Zhangzhou								25		
China	Fujian	Zhangzhou									1	

China	Fujian	Zhangzhou										1
China	Fujian		164									
China	Fujian					4						
China	Jiangxi	Jingdezhen	84									
China	Jiangxi	Jingdezhen		2								
China	Jiangxi	Jingdezhen			5							
China	Jiangxi	Jingdezhen				30						
China	Jiangxi	Jingdezhen						1				
China	Jiangxi	Jingdezhen					1					
China	Jiangxi	Jingdezhen							27			
China	Jiangxi	Jingdezhen								86		
China	Jiangxi	Jingdezhen										1
Japan	Hizen		52									
Japan	Hizen					546						
Japan	Hizen								4			
Japan	Hizen									22		

Cluster 1 1650 – 1800 Definite. We recovered 2,737 sherds that were produced between 1650 and 1800.

Country	Region	Sub-Region	Bowl	Covered Box	Cup	Dish	Jar	Kendi	Lid	Potiche	Tea Pot	Vase	Unknown
Burma	Mon	Martaban	17										
Burma	Mon	Martaban				78							
Burma	Mon	Martaban					140						
Burma	Mon	Martaban											
China	Fujian	Anxi	8										
China	Fujian	Dehua	179										

China	Fujian	Dehua		10									
China	Fujian	Dehua			1								
China	Fujian	Dehua				59							
China	Fujian	Dehua							21				
China	Fujian	Dehua								30			
China	Fujian	Zhangzhou	1975										
China	Fujian	Zhangzhou		1									
China	Fujian	Zhangzhou			1								
China	Fujian	Zhangzhou				237							
China	Fujian	Zhangzhou					8						
China	Fujian	Zhangzhou							14				
China	Fujian	Zhangzhou								28			
China	Fujian	Zhangzhou										1	
China	Fujian	Zhangzhou											1
China	Fujian		164										
China	Fujian					5							
China	Jiangsu	Yixing									5		
China	Jiangxi	Jingdezhen	88										
China	Jiangxi	Jingdezhen		2									
China	Jiangxi	Jingdezhen			5								
China	Jiangxi	Jingdezhen				34							
China	Jiangxi	Jingdezhen						1					
China	Jiangxi	Jingdezhen					1						
China	Jiangxi	Jingdezhen							27				
China	Jiangxi	Jingdezhen								87			
China	Jiangxi	Jingdezhen											1

Japan	Hizen		52										
Japan	Hizen					546							
Japan	Hizen								4				
Japan	Hizen									22			
Netherlands	Limburg	Maastricht	1										
Netherlands	Limburg	Maastricht				13							

Cluster 1 1650 – 1800 Possible. We recovered 3,867 sherds that might have been produced between 1650 and 1800.

CLUSTER 2 TRADE CERAMICS

Country	Region	Sub-Region	Basin	Bowl	Dish	Jar	Jarlet	Kendi	Washer	Unknown
China	Fujian	Cizao				2				
China	Fujian	Dehua		1						
China	Fujian	Tongan		22						
China	Fujian			29						
China	Fujian						1			
China	Fujian							1		
China	Guangdong	Guangzhou	3							
China	Guangdong	Guangzhou				3				
China	Guangdong	Guangzhou								1
China	Guangdong		1							
China	Guangdong			1						
China	Guangdong					35				
China	Jiangxi	Jingdezhen			1					
China	Jiangxi	Jingdezhen		1						
China	Zhejiang	Longquan		22						
China	Zhejiang	Longquan			23					
China	Zhejiang	Longquan					2			
China	Zhejiang	Longquan	1							
China	Zhejiang	Longquan							2	
China	South China					17				

Cluster 2 Pre-1400 Definite. We recovered 169 sherds that date prior to 1400.

Country	Region	Sub-Region	Basin	Bowl	Dish	Jar	Jarlet	Kendi	Saucer	Washer	Unknown
China	Fujian	Cizao				2					
China	Fujian	Dehua		1							
China	Fujian	Tongan		22							
China	Fujian			35							
China	Fujian						1				
China	Fujian							1			
China	Guangdong	Guangzhou	3								
China	Guangdong	Guangzhou				3					
China	Guangdong	Guangzhou									1
China	Guangdong		1								
China	Guangdong			1							
China	Guangdong					35					
China	Jiangxi	Jingdezhen			1						
China	Jiangxi	Jingdezhen		1							
China	Zhejiang	Longquan		27							
China	Zhejiang	Longquan			24						
China	Zhejiang	Longquan				2					
China	Zhejiang	Longquan	1								
China	Zhejiang	Longquan							2		
China	Zhejiang	Longquan								2	
China						17					

Cluster 2 Pre-1400 Possible. We recovered 183 sherds that might have been produced prior to 1400.

Country	Region	Sub-Region	Bowl	Jar
Burma			1	
China	Fujian		1	
China	Fujian	Longquan	2	
China				1
Thailand			1	

Cluster 2 1400 - 1450 Definite. We recovered 6 sherds that were produced between 1400 and 1450.

Country	Region	Sub-Region	Bowl	Dish	Jar	Saucer
Burma			1			
China	Fujian		6			
China	Zhejiang	Longquan	7			
China	Zhejiang	Longquan		2		
China	Zhejiang	Longquan				1
China					3	
Thailand	Sukhothai	Sukhothai	1			
Thailand			1			

Cluster 2 1400 - 1450 Possible. We recovered 22 sherds that might have been produced between 1400 and 1450.

Country	Region	Sub-Region	Bottle Jar	Bowl	Covered Box	Dish	Jar
Burma	Mon	Martaban				2	
Burma	Yangon	Twante		34			
Burma	Yangon	Twante					1
Burma				78			
Burma						42	

China	Fujian			1			
China	Jiangxi	Jingdezhen		22			
China	Jiangxi	Jingdezhen			1		
China	Jiangxi	Jingdezhen				19	
China	Jiangxi	Jingdezhen					1
China	Zhejiang	Longquan		2			
China	Zhejiang	Longquan				1	
China							1
Thailand	Satun	Kalong		1			
Thailand	Singburi						1
Thailand	Sukhothai	Sawankhalok	1				
Thailand	Sukhothai	Sawankhalok		9			
Thailand			8				
Thailand				7			
Thailand					1		
Thailand						10	

Cluster 2 1450 - 1550 Definite. We recovered 243 sherds that were produced between 1450 and 1550. The maximum total sherds that could have been produced between 1450 and 1550 is 245 (no table included).

**Country**	**Region**	**Sub-Region**	**Bowl**	**Covered Box**	**Cup**	**Dish**	**Jar**	**Lid**	**Stem Bowl**	**Unknown**
Burma	Karen	Kaw Don	10							
Burma	Mon	Martaban				1				
Burma	Mon	Martaban					20			
Burma							38			
Burma			13							

China	Fujian	Dehua	1							
China	Fujian	Zhangzhou	44							
China	Fujian	Zhangzhou				163				
China	Fujian	Zhangzhou						1		
China	Fujian	Zhangzhou								3
China	Fujian		111							
China	Fujian					4				
China	Jiangxi	Jingdezhen	26							
China	Jiangxi	Jingdezhen		3						
China	Jiangxi	Jingdezhen							1	
China	Jiangxi	Jingdezhen			2					
China	Jiangxi	Jingdezhen				9				
China	Jiangxi	Jingdezhen			1					
China							1			
Japan	Hizen		2							

Cluster 2 1550 - 1650 Definite. We recovered 454 sherds that were produced between 1550 and 1650.

Country	Region	Sub-Region	Bottle Jar	Bowl	Covered Box	Cup	Dish	Jar	Lid	Potiche	Stem Bowl	Unknown
Burma	Karen	Kaw Don		10								
Burma	Mon	Martaban					1					
Burma	Mon	Martaban						34				
Burma							1					
Burma								87				
Burma				13								
China	Fujian	Dehua		2								

China	Fujian	Dehua			1							
China	Fujian	Dehua				1						
China	Fujian	Dehua							4			
China	Fujian	Dehua								4		
China	Fujian	Zhangzhou		50								
China	Fujian	Zhangzhou					164					
China	Fujian	Zhangzhou							1			
China	Fujian	Zhangzhou										4
China	Fujian			33								
China	Fujian			82			15					
China	Jiangxi	Jingdezhen		27								
China	Jiangxi	Jingdezhen			3							
China	Jiangxi	Jingdezhen									1	
China	Jiangxi	Jingdezhen				4						
China	Jiangxi	Jingdezhen					9					
China								3				
Japan	Hizen			2								
Japan	Hizen						1					
Thailand	Sukhothai	Sawankhalok	1									
Thailand			1									
Thailand					1							

Cluster 2 1550 – 1650 Possible. We recovered 560 sherds that might have been produced between 1550 and 1650.

Country	Region	Sub-Region	Basin	Bottle	Bowl	Covered Box	Cup	Dish	Jar	Lid	Platter	Potiche	Saucer
Burma	Mon	Martaban			1								
Burma	Mon	Martaban							3				
China	Fujian	Dehua			25								
China	Fujian	Dehua						9					
China	Fujian	Dehua										3	
China	Fujian	Zhangzhou			28								
China	Fujian	Zhangzhou						13					
China	Fujian				389								
China	Fujian							40					
China	Fujian								15				
China	Fujian											3	
China	Fujian												
China	Jiangxi	Jingdezhen			34								
China	Jiangxi	Jingdezhen								4			
China	Jiangxi	Jingdezhen					2						
China	Jiangxi	Jingdezhen						12					
China	Jiangxi	Jingdezhen										1	
China									3				
China			1										
Japan	Hizen			1									
Japan	Hizen				31								
Japan	Hizen					3							
Japan	Hizen							132					
Japan	Hizen										2		
Japan	Hizen									1			

Japan	Hizen											4	
Japan	Hizen												1
Vietnam	Hai Duong	Hop Le			13								

Cluster 2 1650 – 1800 Definite. We recovered 774 sherds that were produced between 1650 and 1800.

Country	Region	Sub-Region	Basin	Bottle	Bowl	Covered Box	Cup	Dish	Jar	Lid	Platter	Potiche	Saucer
Burma	Mon	Martaban							17				
Burma	Mon	Martaban			1								
Burma								1					
Burma									49				
China	Fujian	Dehua			31								
China	Fujian	Dehua				1							
China	Fujian	Dehua					1						
China	Fujian	Dehua						14					
China	Fujian	Dehua										7	
China	Fujian	Dehua								4			
China	Fujian	Zhangzhou			32								
China	Fujian	Zhangzhou						18					
China	Fujian				404								
China	Fujian									4			
China	Fujian							67					
China	Fujian								15				
China	Fujian										3		
China	Jiangxi	Jingdezhen			35								
China	Jiangxi	Jingdezhen								4			

China	Jiangxi	Jingdezhen					3						
China	Jiangxi	Jingdezhen						12					
China	Jiangxi	Jingdezhen										1	
China									5				
China			1										
Japan	Hizen			1									
Japan	Hizen				32								
Japan	Hizen					2							
Japan	Hizen							133					
Japan	Hizen										2		
Japan	Hizen									1			
Japan	Hizen											4	
Japan	Hizen												1
Vietnam	Hai Duong	Hop Le			13								

Cluster 2 1650 – 1800 Possible. We recovered 919 sherds that might have been produced between 1650 and 1800.

CLUSTER 3 TRADE CERAMICS

Country	Region	Sub-Region	Basin	Bowl	Dish	Jar	Pot	Unknown
China	Fujian	Cizao				2		
China	Fujian	Tongan		2				
China	Guangdong		15					
China	Guangdong					49		
China	Guangdong						3	
China	Zhejiang	Longquan		2				
China	Zhejiang	Longquan			2			
China	Zhejiang	Longquan						1

Cluster 3 Pre-1400. We recovered 76 sherds that date prior to 1400. We recovered 77 sherds that might date from this period (table not included).

Country	Region	Sub-Region	Bowl
China	Zhejiang	Longquan	1

Cluster 3 1400 – 1450 Definite. We recovered 1 sherd that was produced between 1400 and 1450.

Country	Region	Sub-Region	Bowl	Dish	Jar
Burma	Mon	Martaban	5		
Burma	Mon	Martaban		10	
Burma	Mon	Martaban			23
China	Guangdong				1
China	Zhejiang	Longquan	1		
Thailand	Sukhothai	Sawankhalok		6	

Cluster 3 1400 – 1450 Possible. We recovered 46 sherds that might have been produced between 1400 and 1450.

Country	Region	Sub-Region	Bowl	Covered Box	Dish	Jar	Lid
Burma	Mon	Martaban	5				
Burma	Mon	Martaban			55		
Burma	Mon	Martaban				1	
China	Jiangxi	Jingdezhen	5				
China	Jiangxi	Jingdezhen			34		
China	Jiangxi	Jingdezhen				1	
China	Jiangxi	Jingdezhen					1
Thailand	Sukhothai	Sawankhalok		1			
Thailand	Sukhothai	Sawankhalok			2		
Thailand	Sukhothai	Sawankhalok				1	
Thailand	Sukhothai	Sawankhalok					1

Cluster 3 1450 – 1550 Definite. We recovered 107 sherd that were produced between 1450 and 1550.

Country	Region	Sub-Region	Basin	Bowl	Covered Box	Dish	Jar	Lid
Burma	Mon	Martaban	1					
Burma	Mon	Martaban		13				
Burma	Mon	Martaban				70		
Burma	Mon	Martaban					32	
China	Guangdong						1	
China	Jiangxi	Jingdezhen		5				
China	Jiangxi	Jingdezhen				34		
China	Jiangxi	Jingdezhen					1	
China	Jiangxi	Jingdezhen						1
Thailand	Sukhothai	Sawankhalok		1				

Thailand	Sukhothai	Sawankhalok			1			
Thailand	Sukhothai	Sawankhalok				8		
Thailand	Sukhothai	Sawankhalok					2	
Thailand	Sukhothai	Sawankhalok						1

Cluster 3 1450 – 1550 Possible. We recovered 171 sherds that could have been produced between 1450 and 1550.

Country	Region	Sub-Region	Bowl	Covered Box	Cup	Dish	Jar	Lid	Pot	Potiche	Unknown
Burma	Mon	Martaban	4								
Burma	Mon	Martaban				6					
Burma	Mon	Martaban					56				
Burma	Mon	Martaban							2		
China	Fujian	Dehua	2								
China	Fujian	Dehua				1					
China	Fujian	Zhangzhou	169								
China	Fujian	Zhangzhou				235					
China	Fujian	Zhangzhou					2				
China	Fujian	Zhangzhou						1			
China	Jiangxi	Jingdezhen	70								
China	Jiangxi	Jingdezhen		3							
China	Jiangxi	Jingdezhen			1						
China	Jiangxi	Jingdezhen				30					
China	Jiangxi	Jingdezhen					1				
China	Jiangxi	Jingdezhen						2			
China	Jiangxi	Jingdezhen								3	
China	Jiangxi	Jingdezhen									2

Japan	Hizen					2					

Cluster 3 1550 – 1650 Definite. We recovered 592 sherds that were produced between 1550 and 1650.

Country	Region	Sub-Region	Basin	Bowl	Covered Box	Cup	Dish	Jar	Lid	Pot	Potiche	Vase	Unknown
Burma	Mon	Martaban	1										
Burma	Mon	Martaban		17									
Burma	Mon	Martaban					98						
Burma	Mon	Martaban						256					
Burma	Mon	Martaban								2			
China	Fujian	Dehua		4									
China	Fujian	Dehua			2								
China	Fujian	Dehua					1						
China	Fujian	Dehua							10				
China	Fujian	Dehua									6		
China	Fujian	Shaowu						3					
China	Fujian	Zhangzhou		173									
China	Fujian	Zhangzhou					235						
China	Fujian	Zhangzhou						2					
China	Fujian	Zhangzhou							1				
China	Jiangxi	Jingdezhen		73									
China	Jiangxi	Jingdezhen			3								
China	Jiangxi	Jingdezhen				1							
China	Jiangxi	Jingdezhen					31						
China	Jiangxi	Jingdezhen						1					
China	Jiangxi	Jingdezhen							2				

China	Jiangxi	Jingdezhen									3		
China	Jiangxi	Jingdezhen											2
China	Jiangxi	Jingdezhen										1	
Japan	Hizen						2						
Thailand	Sukhothai	Sawankhalok		1									
Thailand	Sukhothai	Sawankhalok						2					

Cluster 3 1550 – 1650 Possible. We recovered 933 sherds that might have been produced between 1550 and 1650.

Country	Region	Sub-Region	Bowl	Cup	Dish	Jar	Lid	Potiche	Vase	Unknown
Burma	Mon	Martaban			6					
Burma	Mon	Martaban				2				
China	Fujian	Dehua	27							
China	Fujian	Dehua		1						
China	Fujian	Dehua			9					
China	Fujian	Dehua					1			
China	Fujian	Shaowu				1				
China	Fujian	Zhangzhou	307							
China	Fujian	Zhangzhou			38					
China	Fujian	Zhangzhou					6			
China	Fujian	Zhangzhou						1		
China	Fujian		6							
China	Jiangxi	Jingdezhen	38							
China	Jiangxi	Jingdezhen			26					
China	Jiangxi	Jingdezhen					10			
China	Jiangxi	Jingdezhen						18		

China	Jiangxi	Jingdezhen							1	
China	Jiangxi	Jingdezhen								1
China	Zhejiang	Longquan			1					
Japan	Hizen		29							
Japan	Hizen				157					
Japan	Hizen						1			
Japan	Hizen							2		
Vietnam	Hai Duong	Hop Le	38							

Cluster 3 1650 – 1800 Definite. We recovered 727 sherds that were produced between 1650 and 1800.

Country	Region	Sub-Region	Bowl	Covered Box	Cup	Dish	Jar	Lid	Potiche	Tea Pot	Vase	Unknown
Burma	Mon	Martaban	10									
Burma	Mon	Martaban				93						
Burma	Mon	Martaban					194					
China	Fujian	Dehua	47									
China	Fujian	Dehua		2								
China	Fujian	Dehua			1							
China	Fujian	Dehua				18						
China	Fujian	Dehua						11				
China	Fujian	Dehua							6			
China	Fujian	Shaowu					4					
China	Fujian	Zhangzhou	384									
China	Fujian	Zhangzhou				53						
China	Fujian	Zhangzhou						6				
China	Fujian	Zhangzhou							1			

China	Fujian		6									
China	Jiangsu	Yixing								2		
China	Jiangxi	Jingdezhen	41									
China	Jiangxi	Jingdezhen				27						
China	Jiangxi	Jingdezhen						10				
China	Jiangxi	Jingdezhen							18			
China	Jiangxi	Jingdezhen										1
China	Jiangxi	Jingdezhen									2	
China	Zhejiang	Longquan				1						
Japan	Hizen		29									
Japan	Hizen					157						
Japan	Hizen							1				
Japan	Hizen								2			
Thailand	Sukhothai	Sawankhalok				1						
Vietnam	Hai Duong	Hop Le	38									

Cluster 3 1650 – 1800 Possible. We recovered 1,166 sherds that might have been produced between 1650 and 1800.

CLUSTER 4 TRADE CERAMICS

Country	Region	Sub-Region	Bowl	Box	Dish	Jar	Jarlet	Washer	Unknown
China	Fujian	Cizao				1			
China	Fujian	Tongan	3						
China	Fujian		36						
China	Fujian				3				
China	Fujian					5			
China	Fujian						2		
China	Guangdong		2						
China	Guangdong					7			
China	Jiangxi	Jingdezhen	2						
China	Jiangxi	Jingdezhen		1					
China	Jiangxi	Jingdezhen			1				
China	Jiangxi	Jingdezhen				1			
China	Zhejiang	Longquan	36						
China	Zhejiang	Longquan			8				
China	Zhejiang	Longquan				1			
China	Zhejiang	Longquan					1		
China	Zhejiang	Longquan						2	

Cluster 4 Pre-1400 Definite. We recovered 112 sherds that were produced date prior to 1400.

Country	Region	Sub-Region	Bowl	Box	Dish	Jar	Jarlet	Washer
China	Fujian	Cizao						
China	Fujian	Cizao				3		

China	Fujian	Tongan	12					
China	Fujian		36					
China	Fujian				3			
China	Fujian					5		
China	Fujian						2	
China	Guangdong		2					
China	Guangdong					7		
China	Jiangxi	Jingdezhen	2					
China	Jiangxi	Jingdezhen		1				
China	Jiangxi	Jingdezhen			1			
China	Jiangxi	Jingdezhen				1		
China	Zhejiang	Longquan	36					
China	Zhejiang	Longquan			14			
China	Zhejiang	Longquan				1		
China	Zhejiang	Longquan					1	
China	Zhejiang	Longquan						2
Thailand			1					

Cluster 4 Pre-1400 Possible. We recovered 130 sherds that might have been produced prior to 1400.

Country	Region	Sub-Region	Bowl
China	Jiangxi	Jingdezhen	2

Cluster 4 1400 - 1450 Definite. We recovered 2 sherds that were produced between 1400 and 1450.

Country	Region	Sub-Region	Basin	Bowl	Box	Covered Box	Dish	Jar	Unknown
China	Fujian	Tongan		9					
China	Fujian	Zhangzhou		1					
China	Jiangxi	Jingdezhen		2					
China	Zhejiang	Longquan		2					
China	Zhejiang	Longquan					6		
Thailand	Sukhothai	Sawankhalok		10					
Thailand	Sukhothai	Sawankhalok					4		
Thailand	Sukhothai	Sawankhalok							2
Thailand			1						
Thailand				11					
Thailand							12		
Vietnam	Vietnam	Hai Duong			1				
Vietnam	Vietnam	Hai Duong				1			
Vietnam	Vietnam	Hai Duong						1	

Cluster 4 1400 - 1450 Possible. We recovered 63 sherds that might have been produced between 1400 and 1450.

Country	Region	Sub-Region	Bottle	Bowl	Box	Dish	Jar	Lid
China	Fujian	Zhangzhou		1				
China	Fujian			7				
China	Jiangxi	Jingdezhen	1					
China	Jiangxi	Jingdezhen		55				
China	Jiangxi	Jingdezhen			3			
China	Jiangxi	Jingdezhen				52		
China	Jiangxi	Jingdezhen						2

China	Zhejiang	Longquan		26				
China	Zhejiang	Longquan				7		
Thailand	Sukhothai	Sawankhalok		6				
Thailand	Sukhothai	Sawankhalok				17		
Thailand	Sukhothai	Sawankhalok					1	
Thailand				9				
Thailand						11		
Thailand							2	
Vietnam	Hai Duong				1			
Vietnam	Hai Duong						7	

Cluster 4 1450 - 1550 Definite. We recovered 208 sherds that were produced between 1450 and 1550.

Country	Region	Sub-Region	Basin	Bottle	Bowl	Box	Covered Box	Cup	Dish	Jar	Lid	Unknown
Burma	Mon	Martaban								2		
Burma					2							
Burma									2			
Burma										2		
China	Fujian	Zhangzhou			2							
China	Fujian				23							
China	Jiangxi	Jingdezhen		1								
China	Jiangxi	Jingdezhen										
China	Jiangxi	Jingdezhen			95							
China	Jiangxi	Jingdezhen				3						
China	Jiangxi	Jingdezhen						1				
China	Jiangxi	Jingdezhen							56			

China	Jiangxi	Jingdezhen									2	
China	Zhejiang	Longquan			32							
China	Zhejiang	Longquan							10			
China	Zhejiang	Longquan								1		
Thailand	Sukhothai	Sawankhalok		20								
Thailand	Sukhothai	Sawankhalok			1							
Thailand	Sukhothai	Sawankhalok				1						
Thailand	Sukhothai	Sawankhalok						27				
Thailand	Sukhothai	Sawankhalok							1			
Thailand	Sukhothai	Sawankhalok									2	
Thailand			1									
Thailand					30							
Thailand									35			
Thailand										4		
Vietnam	Hai Duong				1							
Vietnam	Hai Duong					4						
Vietnam	Hai Duong								1			
Vietnam	Hai Duong									8		

Cluster 4 1450 - 1550 Possible. We recovered 370 sherds that might have been produced between 1450 and 1550.

Country	Region	Sub-Region	Bottle	Bowl	Box	Cup	Dish	Incence Burner	Jar	Jarlet	Lid	Stem Cup	Unknown
Burma	Mon	Martaban							1				
China	Fujian	Dehua					1						
China	Fujian	Zhangzhou	1										
China	Fujian	Zhangzhou		114									

China	Fujian	Zhangzhou					342						
China	Fujian	Zhangzhou						1					
China	Fujian	Zhangzhou							2				
China	Fujian			11									
China	Fujian						3						
China	Fujian								1				
China	Fujian									1			
China	Fujian												1
China	Jiangxi	Jingdezhen	1										
China	Jiangxi	Jingdezhen		79									
China	Jiangxi	Jingdezhen			1								
China	Jiangxi	Jingdezhen									5		
China	Jiangxi	Jingdezhen				10							
China	Jiangxi	Jingdezhen					12						
China	Jiangxi	Jingdezhen										1	
China	Jiangxi	Jingdezhen											3

Cluster 4 1550 – 1650 Definite. We recovered 591 sherds that were produced between 1550 and 1650.

Country	Region	Sub-Region	Bottle	Bowl	Box	Covered Box	Covered Jar	Cup	Dish	Incense Burner	Jar	Jarlet	Lid	Stem Bowl	Stem Cup	Tray	Vase	Unknown
Burma	Mon	Martaban		9														
Burma	Mon	Martaban							2									
Burma	Mon	Martaban									43							
Burma				2														
Burma									3									
Burma											5							

China	Fujian	Dehua		17														
China	Fujian	Dehua			5													
China	Fujian	Dehua				4												
China	Fujian	Dehua					4											
China	Fujian	Dehua						3										
China	Fujian	Dehua							3									
China	Fujian	Dehua											2					
China	Fujian	Zhangzhou	1															
China	Fujian	Zhangzhou		152														
China	Fujian	Zhangzhou							366									
China	Fujian	Zhangzhou								1								
China	Fujian	Zhangzhou									2							
China	Fujian			173														
China	Fujian							4										
China	Fujian								3									
China	Fujian										23							
China	Fujian											1						
China	Fujian																	3
China	Fujian													2				
China	Guangdong										1							
China	Jiangxi	Jingdezhen	1															
China	Jiangxi	Jingdezhen		171														
China	Jiangxi	Jingdezhen			1													
China	Jiangxi	Jingdezhen				2												
China	Jiangxi	Jingdezhen					1											
China	Jiangxi	Jingdezhen						18										

China	Jiangxi	Jingdezhen							22									
China	Jiangxi	Jingdezhen														1		
China	Jiangxi	Jingdezhen									1							
China	Jiangxi	Jingdezhen															10	
China	Jiangxi	Jingdezhen											5					
China	Jiangxi	Jingdezhen													1			
China	Jiangxi	Jingdezhen																3
China	Zhejiang	Longquan		4														
China	Zhejiang	Longquan							1									
China	Zhejiang	Longquan									1							
Japan	Hizen			4														
Thailand	Sukhothai	Sawankhalok	14															
Thailand	Sukhothai	Sawankhalok		1														
Thailand	Sukhothai	Sawankhalok			1													
Thailand	Sukhothai	Sawankhalok						10										
Thailand	Sukhothai	Sawankhalok															2	
Thailand				19														
Thailand									24									
Thailand											2							
Vietnam	Hai Duong			1														
Vietnam	Hai Duong				2													
Vietnam	Hai Duong								1									

Cluster 4 1550 - 1650 Possible. We recovered 1,158 sherds that might have been produced between 1550 and 1650.

Country	Region	Sub-Region	Basin	Bottle	Bowl	Covered Box	Cup	Dish	Incense Burner	Jar	Lamp	Lid	Pot	Potiche	Spoon	Spoon Tray	Vase	Unknown
Burma	Mon	Martaban								270								
Burma											1							
Burma			8															
Burma				1														
Burma					23													
Burma								44										
Burma										1								
Burma												1						
Burma													1					
China	Fujian	Dehua		1														
China	Fujian	Dehua			168													
China	Fujian	Dehua				2												
China	Fujian	Dehua						23										
China	Fujian	Dehua										5						
China	Fujian	Dehua					3											
China	Fujian	Dehua													1			
China	Fujian	Dehua															3	
China	Fujian	Zhangzhou			286													
China	Fujian	Zhangzhou						127										
China	Fujian	Zhangzhou										2						
China	Fujian	Zhangzhou															1	
China	Fujian		1															
China	Fujian				220													
China	Fujian					1												
China	Fujian							8										

China	Fujian									7								
China	Fujian																	
China	Fujian																	1
China	Fujian						5											
China	Guangdong				5													
China	Jiangxi	Jingdezhen		3														
China	Jiangxi	Jingdezhen			97													
China	Jiangxi	Jingdezhen										8						
China	Jiangxi	Jingdezhen					12											
China	Jiangxi	Jingdezhen						10										
China	Jiangxi	Jingdezhen								5								
China	Jiangxi	Jingdezhen												2				
China	Jiangxi	Jingdezhen														1		
China	Jiangxi	Jingdezhen															1	
Japan	Hizen							1										
Japan	Hizen				19													
Japan	Hizen											4						
Japan	Hizen					2												
Japan	Hizen							291										
Japan	Hizen								1									
Japan	Hizen									5								
Japan	Hizen													6				
Japan	Hizen																	3
Vietnam	Hai Duong	Hop Le			42													
Vietnam	Hai Duong	Hop Le						5										

Cluster 4 1650 - 1800 Definite. We recovered 1,738 sherds that were produced between 1650 and 1800.

Country	Region	Sub-Region	Basin	Bottle	Bowl	Box	Covered Box	Cup	Dish	Incense Burner	Jar	Lamp	Lid	Pot	Potiche	Spoon	Spoon Tray	Stem Cup	Tray	Vase	Unknown
Burma	Mon	Martaban			9																
Burma	Mon	Martaban							2												
Burma	Mon	Martaban									311										
Burma	Mon	Martaban										1									
Burma			8																		
Burma				1																	
Burma					25																
Burma									47												
Burma													1								
Burma														1							
China	Fujian	Dehua		1																	
China	Fujian	Dehua			217																
China	Fujian	Dehua				5															
China	Fujian	Dehua					6														
China	Fujian	Dehua									5										
China	Fujian	Dehua						6													
China	Fujian	Dehua							34												
China	Fujian	Dehua											7								
China	Fujian	Dehua													1						
China	Fujian	Dehua														1					
China	Fujian	Dehua																		3	
China	Fujian	Zhangzhou			442																
China	Fujian	Zhangzhou						1													
China	Fujian	Zhangzhou							260												

China	Fujian	Zhangzhou											4								
China	Fujian	Zhangzhou																		1	
China	Fujian	Zhangzhou																			3
China	Fujian		1																		
China	Fujian				383																
China	Fujian						1														
China	Fujian							9													
China	Fujian								11												
China	Fujian										39										
China	Fujian																				2
China	Fujian															2					
China	Guangdong				6																
China	Guangdong										3										
China	Jiangxi	Jingdezhen		3																	
China	Jiangxi	Jingdezhen			164																
China	Jiangxi	Jingdezhen				1															
China	Jiangxi	Jingdezhen											9								
China	Jiangxi	Jingdezhen					1														
China	Jiangxi	Jingdezhen									7										
China	Jiangxi	Jingdezhen						22													
China	Jiangxi	Jingdezhen							17												
China	Jiangxi	Jingdezhen																	1		
China	Jiangxi	Jingdezhen																		10	
China	Jiangxi	Jingdezhen													2						
China	Jiangxi	Jingdezhen															1				
China	Jiangxi	Jingdezhen																1			

Japan	Hizen								1												
Japan	Hizen				23																
Japan	Hizen												4								
Japan	Hizen						2														
Japan	Hizen								291												
Japan	Hizen									1											
Japan	Hizen										5										
Japan	Hizen														6						
Japan	Hizen																				3
Thailand					4																
Thailand									1												
Vietnam	Hai Duong	Hop Le			42																
Vietnam	Hai Duong	Hop Le							5												

Cluster 4 1650 - 1800 Possible. We recovered 2,487 sherds that might have been produced between 1650 and 1800.

CLUSTER 5 TRADE CERAMICS

Country	Region	Sub-Region	Basin	Bowl	Jar	Jarlet	Kendi	Unknown
China	Fujian	Minnan		1				
China	Fujian	Quanzhou		1				
China	Fujian		2					
China	Fujian			46				
China	Fujian				16			
China	Fujian					2		
China	Fujian							1
China	Guangdong			3				
China	Guangdong				1			
China	Guangxi				2			
China	Zhejiang	Longquan		21				
China	Shannxi	Yaozhou		1				
China				9				
China					17			
China						1		
Thailand							1	

Cluster 5 Pre-1400 Definite. We recovered 125 sherds that were produced prior to 1400.

Country	Region	Sub-Region	Basin	Bowl	Jar	Jarlet	Kendi	Unknown
China	Fujian	Minnan		1				
China	Fujian	Quanzhou		1				
China	Fujian		2					

China	Fujian			50				
China	Fujian				21			
China	Fujian					2		
China	Fujian							1
China	Guangdong			3				
China	Guangdong				1			
China	Guangxi				2			
China	Zhejiang	Longquan		39				
China	Zhejiang	Longquan				1		
China	Shannxi	Yaozhou		1				
China				9				
China					18			
China						1		
China			1					
Thailand							1	

Cluster 5 Pre-1400 Possible. We recovered 155 sherds that might have been produced prior to 1400.

Country	Region	Sub-Region	Basin	Bowl	Jar	Jarlet
China	Fujian			7		
China	Fujian				5	
China	Guangdong			1		
China	Zhejiang	Longquan		19		
China					3	
China			1			
Thailand				1		

Cluster 5 1400 – 1450 Possible. There were no sherds in Cluster 5 that were definitely produced between 1400 and 1450. We recovered 37 sherds that might have been produced between 1400 and 1450.

Country	Region	Sub-Region	Bottle	Bowl	Jar
China	Zhejiang	Longquan		1	
Thailand					2
Thailand				2	

Cluster 5 1450 – 1550 Definite. We recovered 5 sherds that were produced between 1450 and 1550 from this Cluster.

Country	Region	Sub-Region	Basin	Bottle	Bowl	Covered Box	Jar
China	Fujian				14		
China	Fujian						5
China	Guangdong				1		
China	Jiangxi	Jingdezhen			3		
China	Zhejiang	Longquan			1		
China					1		
China							3
China			1				
Thailand				1			
Thailand					3		
Thailand						1	
Thailand							1

Cluster 5 1450 – 1550 Possible. We recovered 35 sherds that might have been produced between 1450 and 1550

Country	Region	Sub-Region	Bowl	Cup	Dish	Lid
China	Fujian	Zhangzhou	1			
China	Fujian		57			
China	Fujian				1	
China	Fujian			1		
China	Jiangxi	Jingdezhen	3			
China	Jiangxi	Jingdezhen				1
China	Jiangxi	Jingdezhen			1	

Cluster 5 1550 – 1650 Definite. We recovered 65 sherds that were produced between 1550 and 1650.

Country	Region	Sub-Region	Basin	Bowl	Covered Box	Cup	Dish	Jar	Lid
Burma	Mon	Martaban		5					
Burma	Mon	Martaban						55	
China	Fujian	Zhangzhou		1					
China	Fujian			169					
China	Fujian				2				
China	Fujian						3		
China	Fujian							1	
China	Fujian		2						
China	Fujian					1			
China	Jiangxi	Jingdezhen		14					
China	Jiangxi	Jingdezhen			1				
China	Jiangxi	Jingdezhen							1
China	Jiangxi	Jingdezhen					3		
China				1					

China								5	
Japan	Hizen			3					
Thailand					1				

Cluster 5 1550 – 1650 Possible. We recovered 268 sherds that might have been produced between 1550 and 1650.

Country	Region	Sub-Region	Bowl	Covered Box	Cup	Dish	Jar	Lid	Plate	Saucer	Spoon	Vase	Unknown
Burma	Mon	Martaban	4										
China	Fujian		525										
China	Fujian							3					
China	Fujian			5									
China	Fujian					2							
China	Fujian						6						
China	Fujian												2
China	Fujian				2								
China	Fujian										1		
China	Fujian											2	
China	Jiangxi	Jingdezhen	18										
China	Jiangxi	Jingdezhen		1									
China	Jiangxi	Jingdezhen							1				
China	Jiangxi	Jingdezhen								1			
China	Jiangxi	Jingdezhen			2								
China			1										
China							2						
Japan	Hizen		28										
Japan	Hizen				2								

Japan	Hizen					20							
Japan	Hizen								7				

Cluster 5 1650 – 1800 Definite. We recovered 635 sherds that were produced between 1650 and 1800.

Country	Region	Sub-Region	Basin	Bowl	Covered Box	Cup	Dish	Jar	Lid	Plate	Spoon	Vase	Unknown
Burma	Mon	Martaban		9									
Burma	Mon	Martaban						55					
China	Fujian			875									
China	Fujian										3		
China	Fujian								3				
China	Fujian				8								
China	Fujian						14						
China	Fujian							7					
China	Fujian		2										
China	Fujian												2
China	Fujian					2							
China	Fujian											2	
China	Jiangxi	Jingdezhen		27									
China	Jiangxi	Jingdezhen			2								
China	Jiangxi	Jingdezhen								1			
China	Jiangxi	Jingdezhen											1
China	Jiangxi	Jingdezhen					3						
China	Jiangxi	Jingdezhen				2							
China				3									
China								10					

Japan	Hizen			34									
Japan	Hizen					2							
Japan	Hizen						21						
Japan	Hizen									7			

Cluster 5 1650 – 1800 Possible. We recovered 1095 sherds that might have been produced between 1650 and 1800.

CLUSTER 6 TRADE CERAMICS DATA

Country	Region	Sub-Region	Bowl	Dish	Jar	Jarlet	Lid
China	Fujian	Cizao			5		
China	Fujian	Tongan	5				
China	Fujian	Tongan		1			
China	Fujian	Zhangzhou		1			
China	Guangdong				44		
China	Guangdong						1
China	Zhejiang	Longquan	4				
China	Zhejiang	Longquan		8			
China	Zhejiang	Longquan			1		
China	Zhejiang	Longquan				1	

Cluster 6 Pre-1400 Definite. This table includes a total of 71 sherds that date prior to 1400 (no overlapping sherds).

Country	Region	Sub-Region	Dish	Jar
Burma	Mon	Martaban	1	
Burma	Mon	Martaban		9
Thailand	Sukhothai	Sawankhalok	2	

Cluster 6 1400 - 1450 Possible. No sherds were recovered from this Cluster that were definitely produced between 1400 and 1450. We recovered 12 sherds that could possibly have been produced during this period.

Country	Region	Sub-Region	Bottle	Bowl	Covered Box	Dish	Jar	Lid
Burma	Mon	Martaban		1				

Burma	Mon	Martaban				14		
Burma	Mon	Martaban					1	
Burma	Yanggong	Twante		1				
China	Jiangxi	Jingdezhen		4				
China	Jiangxi	Jingdezhen				12		
China	Jiangxi	Jingdezhen					3	
Thailand	Sukhothai	Sawankhalok	1					
Thailand	Sukhothai	Sawankhalok			1			
Thailand	Sukhothai	Sawankhalok				1		
Thailand	Sukhothai	Sawankhalok						1
Thailand	Sukhothai	Sukhothai		1				
Thailand	Sukhothai	Sukhothai				4		

Cluster 6 1450 - 1550 Definite. We recovered 45 sherds that were produced between 1450 and 1550.

Country	Region	Sub-Region	Bottle	Bowl	Covered Box	Dish	Jar	Lid	Potiche
Burma	Mon	Martaban		1					
Burma	Mon	Martaban				15			
Burma	Mon	Martaban					10		
Burma	Yanggong	Twante		1					
Burma				4					
China	Jiangxi	Jingdezhen		4					
China	Jiangxi	Jingdezhen				12			
China	Jiangxi	Jingdezhen					3		
Thailand	Sukhothai	Sawankhalok	1						
Thailand	Sukhothai	Sawankhalok		1					
Thailand	Sukhothai	Sawankhalok			1				

Thailand	Sukhothai	Sawankhalok				3			
Thailand	Sukhothai	Sawankhalok					1		
Thailand	Sukhothai	Sawankhalok						1	
Thailand	Sukhothai	Sawankhalok							1
Thailand	Sukhothai	Sukhothai		1					
Thailand	Sukhothai	Sukhothai				4			

Cluster 6 1450 - 1550 Possible. We recovered 64 sherds that could have possibly been produced between 1450 and 1550.

Country	Region	Sub-Region	Bottle	Bowl	Covered Box	Cup	Dish	Jar	Lid	Potiche	Spoon	Stem Cup	Unknown
Burma	Mon	Martaban		3									
Burma	Mon	Martaban					9						
Burma	Mon	Martaban						50					
Burma				2									
Burma							1						
Burma								1					
China	Fujian	Dehua		7									
China	Fujian	Shaowu						2					
China	Fujian	Zhangzhou		365									
China	Fujian	Zhangzhou			3								
China	Fujian	Zhangzhou				1							
China	Fujian	Zhangzhou					592						
China	Fujian	Zhangzhou						1					
China	Fujian	Zhangzhou							2				
China	Fujian	Zhangzhou								2			
China	Fujian	Zhangzhou									1		

China	Fujian	Zhangzhou										1	
China	Fujian	Zhangzhou											1
China	Fujian			1									
China	Jiangxi	Jingdezhen	1										
China	Jiangxi	Jingdezhen		80									
China	Jiangxi	Jingdezhen			1								
China	Jiangxi	Jingdezhen				1							
China	Jiangxi	Jingdezhen					21						
China	Jiangxi	Jingdezhen							2				
China	Jiangxi	Jingdezhen								4			
China	Jiangxi	Jingdezhen										1	
Japan	Hizen			1									

Cluster 6 1550 - 1650 Definite. We recovered 1,157 sherds that were produced between 1550 and 1650.

Country	Region	Sub-Region	Basin	Bottle	Bowl	Covered Box	Cup	Dish	Handle	Jar	Lid	Potiche	Spoon	Stem Cup	Unknown
Burma	Mon	Martaban	1												
Burma	Mon	Martaban			5										
Burma	Mon	Martaban						23							
Burma	Mon	Martaban							1						
Burma	Mon	Martaban								191					
Burma	Mon	Martaban									1				
Burma	Mon	Martaban													1
Burma					6										
Burma								2							
Burma										4					
China	Fujian	Dehua			16										

China	Fujian	Dehua				2									
China	Fujian	Dehua									1				
China	Fujian	Dehua										4			
China	Fujian	Dehua											1		
China	Fujian	Shaowu								6					
China	Fujian	Zhangzhou			376										
China	Fujian	Zhangzhou				3									
China	Fujian	Zhangzhou					1								
China	Fujian	Zhangzhou						596							
China	Fujian	Zhangzhou								1					
China	Fujian	Zhangzhou									2				
China	Fujian	Zhangzhou										2			
China	Fujian	Zhangzhou											1		
China	Fujian	Zhangzhou												1	
China	Fujian	Zhangzhou													1
China	Fujian				1										
China	Guangdong									4					
China	Guangdong										2				
China	Jiangxi	Jingdezhen		1											
China	Jiangxi	Jingdezhen			84										
China	Jiangxi	Jingdezhen				1									
China	Jiangxi	Jingdezhen					1								
China	Jiangxi	Jingdezhen						24							
China	Jiangxi	Jingdezhen									3				
China	Jiangxi	Jingdezhen										5			
China	Jiangxi	Jingdezhen												1	

China	Zhejiang	Longquan							1						
Japan	Hizen				2										
Japan	Hizen								2						
Thailand	Sukhothai	Sawankhalok			1										
Thailand	Sukhothai	Sawankhalok									1				
Thailand	Sukhothai	Sawankhalok											1		

Cluster 6 1550 - 1650 Possible. We recovered 1384 sherds that could have been produced between 1550 and 1650.

Country	Region	Sub-Region	Bottle	Bowl	Cavetto	Covered Box	Cup	Dish	Jar	Lid	Potiche
Burma	Mon	Martaban		3							
Burma	Mon	Martaban						26			
Burma	Mon	Martaban							2		
China	Fujian	Anxi		6							
China	Fujian	Dehua		123							
China	Fujian	Dehua					1				
China	Fujian	Dehua						26			
China	Fujian	Dehua			1						
China	Fujian	Shaowu							1		
China	Fujian	Zhangzhou		932							
China	Fujian	Zhangzhou						272			
China	Fujian	Zhangzhou								4	
China	Fujian	Zhangzhou									4
China	Fujian	Zhangzhou							1		
China	Fujian			21							
China	Fujian							1			
China	Guangdong								2		

China	Jiangxi	Jingdezhen		52							
China	Jiangxi	Jingdezhen				1					
China	Jiangxi	Jingdezhen					2				
China	Jiangxi	Jingdezhen						18			
China	Jiangxi	Jingdezhen								27	
China	Jiangxi	Jingdezhen									20
Japan	Hizen		1								
Japan	Hizen			28							
Japan	Hizen							304			
Vietnam	Hai Duong	Hop Le		202							

Cluster 6 1650 - 1800 Definite. We recovered 2081 sherds that were produced between 1650 and 1800.

Country	Region	Sub-Region	Basin	Bottle	Bowl	Cavetto	Covered Box	Cup	Dish	Handle	Jar	Lid	Potiche	Spoon	Unknown
Burma	Mon	Martaban	1												
Burma	Mon	Martaban			5										
Burma	Mon	Martaban							40						
Burma	Mon	Martaban								1					
Burma	Mon	Martaban									143				
Burma	Mon	Martaban										1			
Burma	Mon	Martaban													1
Burma									1						
Burma											3				
China	Fujian	Anxi			6										
China	Fujian	Dehua			209										
China	Fujian	Dehua					2								

China	Fujian	Dehua						1							
China	Fujian	Dehua							79						
China	Fujian	Dehua										6			
China	Fujian	Dehua											4		
China	Fujian	Dehua												1	
China	Fujian	Dehua				1									
China	Fujian	Shaowu									5				
China	Fujian	Zhangzhou			1728										
China	Fujian	Zhangzhou							383						
China	Fujian	Zhangzhou										5			
China	Fujian	Zhangzhou											7		
China	Fujian	Zhangzhou												1	
China	Fujian	Zhangzhou									1				
China	Fujian	Zhangzhou													1
China	Fujian				25										
China	Fujian								1						
China	Guangdong										6				
China	Guangdong											2			
China	Jiangsu	Yixing			1										
China	Jiangxi	Jingdezhen			57										
China	Jiangxi	Jingdezhen					1								
China	Jiangxi	Jingdezhen						2							
China	Jiangxi	Jingdezhen							21						
China	Jiangxi	Jingdezhen										28			
China	Jiangxi	Jingdezhen											22		
China	Zhejiang	Longquan							1						

Holland	Limburg	Maastricht			2										
Holland	Limburg	Maastricht													
Japan	Hizen			1											
Japan	Hizen				30										
Japan	Hizen								306						
Vietnam	Hai Duong	Hop Le			203										

Cluster 6 1650 - 1800 Possible. We recovered 3345 sherds that could have been produced between 1650 and 1800.

CLUSTER 7 TRADE CERAMICS DATA

Country	Region	Sub-Region	Basin	Bowl	Brush Washer	Covered Box	Dish	Jar	Khadai
China	Fujian	Cizao						10	
China	Fujian	Dehua					1		
China	Fujian	Tongan		54					
China	Fujian			82					
China	Fujian					2			
China	Fujian						1		
China	Fujian							15	
China	Fujian		1						
China	Guangdong	Guangzhou	10						
China	Guangdong	Guangzhou						4	
China	Guangdong							4	
China	Guangdong			1					
China	Zhejiang	Longquan		13					
China	Zhejiang	Longquan			1				
China	Zhejiang	Longquan					8		
China				1					
China								11	
India									1

Cluster 7 Pre-1400 Definite. This table includes a total of 220 sherds that date prior to 1400.

Country	Region	Sub-Region	Basin	Bowl	Brush Washer	Covered Box	Dish	Jar	Khadai	Vase
China	Fujian	Cizao						10		

China	Fujian	Dehua					1			
China	Fujian	Tongan		54						
China	Fujian			80						
China	Fujian					2				
China	Fujian						5			
China	Fujian							11		
China	Fujian		1							
China	Guangdong	Guangzhou	10							
China	Guangdong	Guangzhou						4		
China	Guangdong							4		
China	Guangdong		1							
China	Guangdong			1						
China	Henan	Yuzhou								1
China	Zhejiang	Longquan		26						
China	Zhejiang	Longquan			1					
China	Zhejiang	Longquan					8			
China				1						
China			1							
China								12		
Thailand				6						
India									1	

Cluster 7 Pre-1400 Possible. This table includes a total of 241 sherds that could possibly date prior to 1400 when all overlapping sherds on included.

Country	Region	Sub-Region	Basin	Bowl
China	Zhejiang	Longquan	1	
China	Zhejiang	Longquan		1

Cluster 7 1400- 1450 Definite. We recovered 2 sherds that were produced between 1400 and 1450.

Country	Region	Sub-Region	Bowl	Bottle	Lid	Basin	Jar	Vase
Burma			3					
China	Fujian		8					
China	Guangdong					1		
China	Henan	Yuzhou						1
China	Zhejiang	Longquan	13					
China	Zhejiang	Longquan				1		
China						1		
China							1	
Thailand				1				
Thailand					1			
Thailand			9					

Cluster 7 1400- 1450 Possible. We recovered 40 sherds that could have been produced between 1400 and 1450.

Country	Region	Sub-Region	Bowl	Dish
Burma	Mon	Martaban	1	
China	Fujian		1	
China	Jiangxi	Jingdezhen	15	
China	Jiangxi	Jingdezhen		2
China	Zhejiang	Longquan	1	

Thailand				1
Vietnam	Hai Duong		1	

Cluster 7 1450- 1550 Definite. We recovered 22 sherds that were produced between 1450 and 1550.

Country	Region	Sub-Region	Bowl	Bottle	Cup	Dish	Jar	Lid	Vase
Burma	Mon	Martaban	1						
Burma			3						
China	Fujian		5						
China	Henan	Yuzhou							1
China	Jiangxi	Jingdezhen	18						
China	Jiangxi	Jingdezhen			2				
China	Jiangxi	Jingdezhen				8			
China	Zhejiang	Longquan	1						
China							1		
Thailand				1					
Thailand								1	
Thailand			7						
Thailand						1			
Vietnam	Hai Duong		1						

Cluster 7 1450- 1550 Possible. We recovered 51 sherds that could have been produced between 1450 and 1550.

Country	Region	Sub-Region	Bottle	Bowl	Covered Box	Cup	Dish	Jar	Lid	Plate	Vase
Burma	Mon	Martaban						7			
Burma	Mon	Martaban		6							
China	Fujian	Dehua		1							

China	Fujian	Dehua						1			
China	Fujian	Zhangzhou	1								
China	Fujian	Zhangzhou		54							
China	Fujian	Zhangzhou			2						
China	Fujian	Zhangzhou					197				
China	Fujian			277							
China	Fujian				2						
China	Fujian						6				
China	Fujian							1			
China	Fujian					1					
China	Jiangxi	Jingdezhen		19							
China	Jiangxi	Jingdezhen			2						
China	Jiangxi	Jingdezhen					6				
China	Jiangxi	Jingdezhen								1	
China	Jiangxi	Jingdezhen									1
China								2			

Cluster 7 1550- 1650 Definite. We recovered 587 sherds that were produced between 1550 and 1650.

Country	Region	Sub-Region	Bottle	Bowl	Covered Box	Cup	Dish	Jar	Lid	Plate	Potiche	Vase	Unknown
Burma	Mon	Martaban						14					
Burma	Mon	Martaban		6									
Burma								2					
China	Fujian	Dehua	1										
China	Fujian	Dehua		3									
China	Fujian	Dehua			3								

China	Fujian	Dehua				3							
China	Fujian	Dehua						1					
China	Fujian	Dehua							2				
China	Fujian	Dehua									7		
China	Fujian	Zhangzhou	1										
China	Fujian	Zhangzhou		66									
China	Fujian	Zhangzhou			2								
China	Fujian	Zhangzhou					213						
China	Fujian			320									
China	Fujian				1								
China	Fujian						9						
China	Fujian							1					
China	Fujian						3						
China	Fujian							1					
China	Fujian								1				
China	Fujian					2							
China	Jiangxi	Jingdezhen		25									
China	Jiangxi	Jingdezhen			2								
China	Jiangxi	Jingdezhen				2							
China	Jiangxi	Jingdezhen					12						
China	Jiangxi	Jingdezhen								1			
China	Jiangxi	Jingdezhen										1	
China	Fujian	Zhangzhou					2						
China								5					
China													1
Japan	Hizen			1									

Thailand				1									

Cluster 7 1550- 1650 Possible. We recovered 715 sherds that were produced between 1550 and 1650.

Country	Region	Sub-Region	Base	Basin	Bowl	Covered Box	Cup	Dish	Jar	Kendi	Lid	Pot	Potiche	Saucer	Spoon	Stem Cup	Vase	Unknown
Burma	Mon	Martaban							269									
Burma	Mon	Martaban			25													
Burma	Mon	Martaban						1										
Burma	Mon	Martaban										1						
Burma					4													
Burma								8										
Burma																		1
China	Fujian	Anxi			2													
China	Fujian	Dehua			63													
China	Fujian	Dehua				3												
China	Fujian	Dehua						13										
China	Fujian	Dehua									2							
China	Fujian	Dehua											3					
China	Fujian	Zhangzhou			135													
China	Fujian	Zhangzhou						98										
China	Fujian	Zhangzhou									1							
China	Fujian				1134													
China	Fujian										9							
China	Fujian					4												
China	Fujian						2											
China	Fujian							121										

China	Fujian								6									
China	Fujian									1								
China	Fujian												6					
China	Fujian															2		
China	Fujian														1			
China	Jiangxi	Jingdezhen			29													
China	Jiangxi	Jingdezhen									3							
China	Jiangxi	Jingdezhen				2												
China	Jiangxi	Jingdezhen					6											
China	Jiangxi	Jingdezhen						19										
China	Jiangxi	Jingdezhen											1					
China	Jiangxi	Jingdezhen												1				
China	Jiangxi	Jingdezhen																1
China									3									
Japan	Hizen							4										
Japan	Hizen		2															
Japan	Hizen				51													
Japan	Hizen										12							
Japan	Hizen					3												
Japan	Hizen							231										
Japan	Hizen			1														
Japan	Hizen												8					
Japan	Hizen													2				
Japan	Hizen																1	
Vietnam	Hai Duong	Hop Le			97													
Vietnam	Hai Duong	Hop Le						33										

Vietnam	Hai Duong								1									

Cluster 7 1650- 1800 Definite. We recovered 2,426 sherds that were produced between 1650 and 1800.

CLUSTER 8 TRADE CERAMICS DATA

Country	Region	Sub-Region	Basin	Bowl	Covered Box	Dish	Ewer	Jar	Potiche	Washer
China	Fujian	Cizao						27		
China	Fujian	Dehua			1					
China	Fujian	Minnan		23						
China	Fujian	Minnan						4		
China	Fujian	Tongan		45						
China	Fujian	Tongan					2			
China	Fujian			1						
China	Fujian							4		
China	Guangdong		7							
China	Guangdong							266		
China	Guangdong	Xicun		1						
China	Guangdong	Xicun	1							
China	Jiangxi	Jingdezhen				1				
China	Zhejiang	Longquan		38						
China	Zhejiang	Longquan				28				
China	Zhejiang	Longquan						1		
China	Zhejiang	Longquan							1	
China	Zhejiang	Longquan								3
China								3		

Cluster 8 Pre-1400 Definite. This table includes a total of 457 sherds that date prior to 1400. When overlapping sherds are included there is a total of 458 sherds that could possibly date to this period (no table included for maximum possible due to negligible difference in sherd count).

Country	Region	Sub-Region	Jar
China	Guangdong		1

Cluster 8 1400 - 1450 Definite. There is 1 sherd that was produced between 1400 and 1450.

Country	Region	Sub-Region	Dish	Jar
Burma	Mon	Martaban	1	
Burma	Mon	Martaban		2
China	Guangdong			1
Thailand	Sukhothai	Sawankhalok	1	
Thailand			2	
Vietnam	Hai Duong		1	

Cluster 8 1400 – 1450 Possible. There are 8 sherds that could have been produced between 1400 and 1450.

Country	Region	Sub-Region	Bowl	Dish	Jar
Burma	Mon	Martaban		2	
Burma	Mon	Martaban			3
Burma	Yangon	Twante	14		
China	Jiangxi	Jingdezhen		2	
Thailand	Sukhothai	Sawankhalok	19		
Vietnam	Hai Duong		1		

Cluster 8 1450 - 1550 Definite. We recovered 41 sherds that were produced between 1450 and 1550.

Region	Sub-Region	Basin	Bowl	Cup	Dish	Jar	Potiche	Unknown
Mon	Martaban	2						
Mon	Martaban				3			
Mon	Martaban					11		
Fujian	Dehua						4	
Fujian	Dehua							1
Fujian	Zhangzhou		12					
Fujian	Zhangzhou				33			
Jiangxi	Jingdezhen		2					
Jiangxi	Jingdezhen			1				
Jiangxi	Jingdezhen				2			
Sukhothai	Sawankhalok		1					

Cluster 8 1450 - 1550 Possible. We recovered 72 sherds that could have been produced between 1450 and 1550.

Country	Region	Sub-Region	Bowl	Dish
China	Fujian	Zhangzhou	12	
China	Fujian	Zhangzhou		32
China	Jiangxi	Jingdezhen	2	
China	Jiangxi	Jingdezhen		2

Cluster 8 1550 - 1650 Definite. We recovered 48 sherds that were produced between 1550 and 1650.

Country	Region	Sub-Region	Basin	Bowl	Cup	Dish	Jar	Potiche	Unknown
Burma	Mon	Martaban	2						
Burma	Mon	Martaban				3			
Burma	Mon	Martaban					11		

China	Fujian	Dehua						4	
China	Fujian	Dehua							1
China	Fujian	Zhangzhou		12					
China	Fujian	Zhangzhou				33			
China	Jiangxi	Jingdezhen		2					
China	Jiangxi	Jingdezhen				2			
Thailand	Sukhothai	Sawankhalok		1					

Cluster 8 1550 - 1650 Possible. We recovered 71 sherds that could have been produced between 1550 and 1650.

Country	Region	Sub-Region	Bowl	Dish
China	Fujian	Dehua	8	
China	Fujian	Zhangzhou	42	
China	Fujian	Zhangzhou		18
China	Fujian		15	
China	Jiangxi	Jingdezhen	1	
Japan	Hizen		2	
Japan	Hizen			6

Cluster 8 1650 - 1800 Definite. We recovered 92 sherds that were produced between 1650 and 1800.

Country	Region	Sub-Region	Basin	Bowl	Cup	Dish	Jar	Potiche	Tea Pot	Unknown
Burma	Mon	Martaban	2							
Burma	Mon	Martaban				3				
Burma	Mon	Martaban					10			
China	Fujian	Dehua		9						
China	Fujian	Dehua				4				

China	Fujian	Dehua						4		
China	Fujian	Dehua								1
China	Fujian	Zhangzhou		58						
China	Fujian	Zhangzhou				22				
China	Fujian			19						
China	Jiangsu	Yixing							1	
China	Jiangxi	Jingdezhen		1						
China	Jiangxi	Jingdezhen			1					
Japan	Hizen			2						
Japan	Hizen					6				

Cluster 8 1650 - 1800 Possible. We recovered 143 sherds that could have been produced between 1650 and 1800.

CLUSTER 9 TRADE CERAMICS DATA

Country	Region	Sub-Region	Basin	Bottle	Bowl	Jar	Jarlet	Pot	Washer
China	Fujian	Cizao				36			
China	Fujian	Dehua			2				
China	Fujian	Minnan		1					
China	Fujian	Minnan			16				
China	Fujian	Minnan				10			
China	Fujian	Minnan					1		
China	Fujian	Minnan	2						
China	Fujian	Quanzhou			7				
China	Fujian	Tongan			8				
China	Fujian					11			
China	Guangdong					47			
China	Guangdong							1	
China	Zhejiang	Longquan			24				
China	Zhejiang	Longquan							1
China				2					
China						26			
China			1						
Thailand	Sukhothai	Sukhothai			1				

Cluster 9 Pre-1400 Definite. This table includes a total of 197 sherds that date prior to 1400.

Country	Region	Sub-Region	Dish	Jar
Burma	Mon	Martaban	1	
Burma	Mon	Martaban		1

Cluster 9 1400 – 1450 Possible. There are no sherds that were definitely produced between 1400 and 1450. We identified 2 sherds that could have been produced during this period.

Country	Region	Sub-Region	Bowl
Burma	Karen	Kaw Don	2

Cluster 9 1450 – 1550 Definite. Only 2 sherds were recovered that were produced between 1450 and 1550.

Country	Region	Sub-Region	Bowl	Dish	Jar
Burma	Karen	Kaw Don	2		
Burma	Mon	Martaban		2	
Burma	Mon	Martaban			1

Cluster 9 1450 – 1550 Possible. Only 5 sherds were recovered that could have been produced between 1450 and 1550.

Country	Region	Sub-Region	Bowl	Dish
China	Fujian	Zhangzhou	6	
China	Fujian	Zhangzhou		2

Cluster 9 1550 – 1650 Definite. Only 2 sherds were recovered that were produced between 1550 and 1650.

Country	Region	Sub-Region	Bowl	Dish	Jar
Burma	Mon	Martaban		1	
Burma	Mon	Martaban			12
China	Fujian	Zhangzhou	6		
China	Fujian	Zhangzhou		2	

Cluster 9 1550 – 1650 Possible. We recovered 21 sherds that could have been produced between 1550 and 1650.

Country	Region	Sub-Region	Bowl	Dish	Lid	Potiche	Tea Pot
China	Fujian	Dehua	6				
China	Fujian	Dehua		1			
China	Fujian	Zhangzhou	53				
China	Fujian	Zhangzhou		3			
China	Fujian		15				
China	Jiangsu	Yixing					2
China	Jiangxi	Jingdezhen	1				
China	Jiangxi	Jingdezhen			1		
China	Jiangxi	Jingdezhen				1	
Japan	Hizen		1				
Japan	Hizen			1			
Vietnam	Hai Duong	Hop Le	4				

Cluster 9 1650 – 1800 Definite. We recovered 89 sherds that were produced between 1650 and 1800.

Country	Region	Sub-Region	Bowl	Dish	Jar	Lid	Potiche	Tea Pot
Burma	Mon	Martaban			12			
China	Fujian	Dehua	7					
China	Fujian	Dehua		1				
China	Fujian	Zhangzhou	62					
China	Fujian	Zhangzhou		7				
China	Fujian		17					
China	Jiangsu	Yixing						2
China	Jiangxi	Jingdezhen	1					
China	Jiangxi	Jingdezhen				1		
China	Jiangxi	Jingdezhen					1	
Japan	Hizen		1					
Japan	Hizen			1				
Vietnam	Hai Duong	Hop Le	4					

Cluster 9 1650 – 1800 Possible. We recovered 117 sherds that could have been produced between 1650 and 1800.

CLUSTER 10 TRADE CERAMICS DATA

Country	Region	Sub-Region	Basin	Bowl	Box	Brush Washer	Cooking Pot	Covered Box	Dish	Ewer	Jar	Jarlet	Kendi	Khadai	Lid	Pot	Potiche	Saucer	Vase	Unknown
Burma				2																
Burma	Mon	Martaban									2									
China	Fujian	Cizao									70									
China	Fujian	Cizao											1							
China	Fujian	Dehua		4																
China	Fujian	Dehua			4															
China	Fujian	Dehua						6												
China	Fujian	Dehua									1									
China	Fujian	Dehua													1					
China	Fujian	Quanzhou		12																
China	Fujian	Tongan		171																
China	Fujian	Tongan																1		
China	Fujian	Tongan																		1
China	Fujian	Minnan	6																	
China	Fujian	Minnan		141																
China	Fujian	Minnan								1										
China	Fujian	Minnan									8									
China	Fujian	Minnan										3								
China	Fujian		5																	
China	Fujian										21									
China	Fujian			3																
China	Guangdong	Guangzhou									4									
China	Guangdong		37																	
China	Guangdong			15																
China	Guangdong																			
China	Guangdong										1097									
China	Guangdong											2								
China	Guangdong																			4

China	Hebei	Cizhou		1																
China	Hebei	Cizhou									3									
China	Hebei	Quyang		1																
China	Jiangxi	Jingdezhen		68																
China	Jiangxi	Jingdezhen									2									
China	Jiangxi	Jingdezhen							6											
China	Jiangxi	Jingdezhen								2										
China	Jiangxi	Jingdezhen										2								
China	Jiangxi	Jingdezhen																		1
China	Jiangxi	Jingdezhen																	1	
China	Zhejiang	Longquan		260																
China	Zhejiang	Longquan				4														
China	Zhejiang	Longquan							132											
China	Zhejiang	Longquan								1										
China	Zhejiang	Longquan									8									
China	Zhejiang	Longquan										10								
China	Zhejiang	Longquan															1			
China	Zhejiang	Longquan													1					
China											6									
India							9													
India											1									
India														3						
India																				26
India				1																
Syria	Raqqa										1									
Thailand	Ayutthaya															2				
Thailand	Ayutthaya																			2
Vietnam	Hai Duong			9																

Cluster 10 Pre-1400 Definite. This table includes a total of 2,188 sherds that date prior to 1400. This excludes all sherds that extend beyond 1400. A total of 2,221 sherds could possibly date prior to 1400, if all overlapping sherds are included. The difference of 33 sherds has no impact on our analysis and interpretation for this paper, and therefore we do not include a table for all possible sherds from this period.

Country	Region	Sub-Region	Bowl	Covered Box	Dish	Jar	Unknown
Burma			8				
Burma						2	
China	Guangdong					4	
China	Zhejiang	Longquan	3				
China	Zhejiang	Longquan			12		
China	Zhejiang	Longquan	1				
China	Zhejiang	Longquan				1	
Thailand	Ayutthaya						2
Thailand	Sukhothai	Sawankhalok	15				
Thailand	Sukhothai	Sukhothai	1				
Vietnam	Hai Duong		6				
Vietnam	Hai Duong			1			

Cluster 10 1400 - 1450 Definite. We identified a total of 56 sherds that were produced between 1400 – 1450.

Country	Region	Sub-Region	Bottle	Bowl	Covered Box	Dish	Jar	Lid	Unknown
Burma				8					
Burma							2		
Burma	Mon	Martaban		8					
Burma	Mon	Martaban				33			
Burma	Mon	Martaban					105		
Burma	Yangon	Twante		5					
China	Guangdong						5		
China	Zhejiang	Longquan		6					
China	Zhejiang	Longquan				15			

Thailand	Ayutthaya								2
Thailand	Sukhothai	Sawankhalok	3						
Thailand	Sukhothai	Sawankhalok		56					
Thailand	Sukhothai	Sawankhalok				57			
Thailand	Sukhothai	Sawankhalok						4	
Thailand	Sukhothai	Sawankhalok							1
Thailand	Sukhothai	Sukhothai				8			
Thailand	Sukhothai	Sukhothai		1					
Thailand				2					
Vietnam	Hai Duong			15					
Vietnam	Hai Duong				1				
Vietnam	Hai Duong					1			

Cluster 10 1400 - 1450 Possible. We identified a total of 338 sherds that could have been produced between 1400 and 1450. Some of the ceramics from Thailand and Burma can only be dated between 1400 and 1500.

Country	Region	Sub-Region	Basin	Bottle	Bowl	Dish	Jar	Jarlet	Vase
Burma					21				
Burma							4		
Burma	Mon	Martaban	1						
Burma	Mon	Martaban			2				
Burma	Mon	Martaban				5			
Burma	Mon	Martaban					26		
Burma	Yangon	Twante			12				
Burma	Yangon	Twante				1			
China	Fujian				1				

China	Jiangxi	Jingdezhen			9				
China	Jiangxi	Jingdezhen				1			
Thailand	Sukhothai	Sawankhalok		9					
Thailand	Sukhothai	Sawankhalok			102				
Thailand	Sukhothai	Sawankhalok				14			
Thailand	Sukhothai	Sawankhalok						1	
Thailand	Sukhothai	Sawankhalok							1

Cluster 10 1450 - 1550 Definite. We identified a total of 210 sherds that were produced between 1450 and 1550.

Country	Region	Sub-Region	Basin	Bottle	Bowl	Dish	Jar	Jarlet	Lid	Vase	Unknown
Burma	Kaw Don				2						
Burma	Kaw Don						1				
Burma	Mon	Martaban					132				
Burma	Mon	Martaban	1								
Burma	Mon	Martaban			10						
Burma	Mon	Martaban				38					
Burma	Yangon	Twante			17						
Burma	Yangon	Twante				1					
Burma					19						
Burma							4				
China	Fujian				1						
China	Jiangxi	Jingdezhen			10						
China	Jiangxi	Jingdezhen				1					
China	Zhejiang	Longquan			2						
China	Zhejiang	Longquan				3					

Thailand	Sukhothai	Sawankhalok		12							
Thailand	Sukhothai	Sawankhalok			143						
Thailand	Sukhothai	Sawankhalok				71					
Thailand	Sukhothai	Sawankhalok					1				
Thailand	Sukhothai	Sawankhalok							4		
Thailand	Sukhothai	Sawankhalok									1
Thailand	Sukhothai	Sawankhalok								1	
Thailand	Sukhothai	Sukhothai				8					
Thailand					2						
Vietnam	Hai Duong				9						
Vietnam	Hai Duong					1					

Cluster 10 1450 - 1550 Possible. We identified a total of 495 sherds that could have been produced between 1450 – 1550.

Country	Region	Sub-Region	Bowl	Dish
China	Jiangxi	Jingdezhen	6	
China	Jiangxi	Jingdezhen		1

Cluster 10 1550 - 1650 Definite. We identified a total of 7 sherds that were produced between 1550 and 1650.

Country	Region	Sub-Region	Bowl	Jar	Dish
Burma	Mon	Martaban		2	
Burma		Kaw Don		1	
Burma				7	
China	Fujian	Zhangzhou			1
China	Jiangxi	Jingdezhen	7		

Cluster 10 1550 - 1650 Possible. We identified a total of 18 sherds that could have been produced between 1550 and 1650.
